# Marine-Derived *Penicillium* Species as Producers of Cytotoxic Metabolites

**DOI:** 10.3390/md15100329

**Published:** 2017-10-24

**Authors:** Sen Liu, Mingzhi Su, Shao-Jiang Song, Jee H. Jung

**Affiliations:** 1 College of Pharmacy, Pusan National University, Busan 609-735, Korea; ls691392008@sina.com (S.L.); sumingzhi0310@gmail.com (M.S.); 2 Department of Natural Products Chemistry, Shenyang Pharmaceutical University, Shenyang 110016, China; songsj99@163.com

**Keywords:** marine-derived *Penicillium*, natural products, cytotoxic metabolites, biosynthesis

## Abstract

Since the discovery of penicillin, *Penicillium* has become one of the most attractive fungal genera for the production of bioactive molecules. Marine-derived *Penicillium* has provided numerous excellent pharmaceutical leads over the past decades. In this review, we focused on the cytotoxic metabolites * (* Cytotoxic potency was referred to five different levels in this review, extraordinary (IC_50_/LD_50_: <1 μM or 0.5 μg/mL); significant (IC_50_/LD_50_: 1~10 μM or 0.5~5 μg/mL); moderate (IC_50_/LD_50_: 10~30 μM or 5~15 μg/mL); mild (IC_50_/LD_50_: 30~50 μM or 15~25 μg/mL); weak (IC_50_/LD_50_: 50~100 μM or 25~50 μg/mL). The comparative potencies of positive controls were referred when they were available). produced by marine-derived *Penicillium* species, and on their cytotoxicity mechanisms, biosyntheses, and chemical syntheses.

## 1. Introduction

The oceans, which occupy more than 70% of the earth’s surface, undoubtedly support vast habitats and serve as prolific resources of various living organisms. Compared to terrestrial organisms, marine organisms often produce highly potent metabolites with unique structures to enable them to adapt to extremely challenging environments [1]. Developments and improvements made in biotechnology have led to a new era of bioprospecting for new marine products. Revolutionary target screening methods have improved the efficiency of drug discovery. In addition, leading edge genomics of biological symbiosis offer more opportunities to discover drug candidates and precursors. Marine endozoic microorganisms represent a new frontier in the discovery of pharmaceutical agents [2]. In particular, marine-derived fungi are excellent producers of biologically active secondary metabolites. Since the isolation of the broad-spectrum antibiotic, cephalosporin C from the marine-derived fungus *Acremonium chrysogenum*, thousands of bioactive metabolites have been discovered and evaluated [3].

Cancer is the second leading cause of death. Lung, prostate, colorectal, and digestive tract cancer are commonly encountered in males, whereas breast, lung, and cervical cancer are the major causes of female death. Marine microorganisms produce limited amounts of highly efficient toxic substances to protect their hosts from enemies, and these substances have been investigated as potential anticancer drug precursors. In particular, marine-derived *Penicillium* species represent a major source of cytotoxic metabolites. In this review, we list all cytotoxic or antitumor secondary metabolites isolated from marine-derived *Penicillium* species and classify them into distinct chemical groups. In addition, we summarize the cytotoxicity mechanisms and proposed biosyntheses of these metabolites. Overall, more than 200 natural products and their synthetic analogues are included in this review.

## 2. Alkaloids

**Figure 1:**
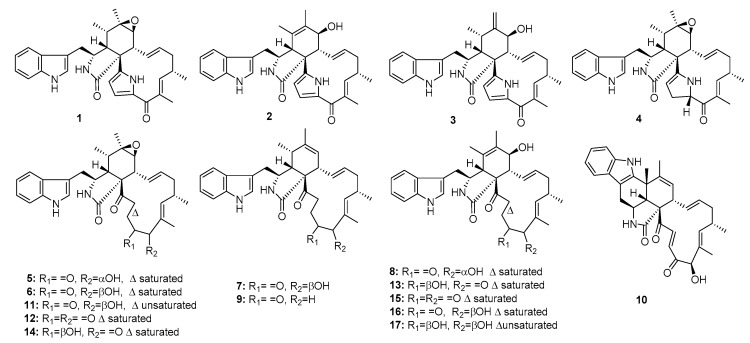
Chemical structures of compounds **1**–**17**.

Cytochalasan alkaloids, characterized by a highly substituted perhydoisoindol-1-one fused to a macrocyclic ring, have been shown to possess potential cytotoxicity against diverse tumor cell lines [4,5]. Penochalasins, chaetoglobosins, and cytoglobosins are common classes of cytochalasan alkaloids. A series of cytochalasans, penochalasins A–J (**1**–**10**), chaetoglobosins A, C, E–G, O (**11**–**16**), and cytoglobosin C (**17**) (Figure 1) were isolated from the mangrove endophytic fungus *P. chrysogenum* [6] and from the marine alga *Enteromorpha* intestinalis [7,8]. Penochalasins A–H (**1**–**8**) and chaetoglobosins A, F, O (**11**, **14**, **16**) exhibited significant cytotoxic activity (ED_50_ = 0.4, 0.3, 0.5, 3.2, 2.1, 1.8, 1.9, 2.8, 0.6, 0.9, and 2.4 μg/mL, respectively) against P388 lymphocytic leukemia cells. Moreover, chaetoglobosin A (**11**) reportedly induced apoptosis of chronic lymphocytic leukemia (CLL) cells by targeting the cytoskeleton. The underlying mechanisms involve the induction of cell-cycle arrest and the inhibition of membrane ruffling and cell migration; therefore, it was proposed as a novel drug for CLL [9]. Penochalasin I (**9**) exhibited significant cytotoxic activities against MDA-MB-435 (human breast cancer cell line) and SGC-7901 (human gastric cancer cell line) with IC_50_ values of ~7 μM. Cytoglobosin C (**17**) showed potential cytotoxicity against both SGC-7901 and A549 (human lung adenocarcinoma) with IC_50_ values of 3–8 μM. Other cytochalasans, penochalasin J (**10**), chaetoglobosins C, E (**12**, **13**), and chaetoglobosin G (**15**) showed moderate cytotoxicity against MDA-MB-435, SGC-7901, and A549 with IC_50_ values in the range of 10–40 μM (epirubicin was used as a positive control with IC_50_ values of 0.3~0.6 μM). A recent biosynthetic analysis showed that the fungal PKS-NRPS hybrid synthase, CheA, plays an essential role in cytochalasan formation [10].

**Figure 2:**
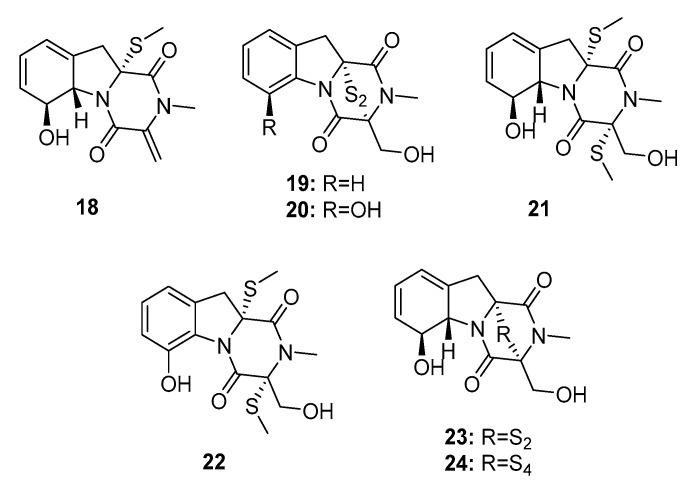
Chemical structures of compounds **18**–**24**.

Gliotoxin induces cellular immunosuppression and apoptosis [11], and its analogues are disulfur or polysulfur-containing mycotoxins that belong to a class of naturally occurring epipolythio piperazines (ETP). In 2012, the marine fungus *Penicillium* sp. JMF034, which was isolated from a deep sea sediment in Japan, was found to produce seven gliotoxin-related compounds, (bis(dethio)-10a-methylthio-3a-deoxy-3,3a-didehydrogliotoxin (**18**), 6-deoxy-5a,6-dide hydrogliotoxin (**19**), bis(dethio) bis(methylthio)gliotoxin (**20**), bis(dethio)bis(methylthio)-5a,6-dide hydrogliotoxin (**21**), 5a,6-dide hydrogliotoxin (**22**), gliotoxin (**23**), and gliotoxin G (**24**) (Figure 2) [12], which potently killed P388 murine leukemia cells (IC_50_ = 3.4, 0.058, 0.11, 0.11, 0.056, 0.024, and 0.020 μM, respectively). Because of their extraordinary cytotoxicity, gliotoxin analogues are considered as antitumor leads [13]. Dimeric ETPs were reported to inhibit histone methyltransferase (HMT); in addition, compounds (**22**–**24**) with disulfide or tetrasulfide bonds showed significant inhibitory activities against HMT G9a (IC_50_ = 2.6, 6.4, and 2.1 μM, respectively) rather than HMT SET7/9 (IC_50_ > 100 μM). Gliotoxin G (**24**), isolated from the mangrove endophytic fungus *P. brocae* MA-231, was potently active against cisplatin-sensitive and resistant human ovarian cancer cell lines, A2780 and A2780 CisR, with IC_50_ values of 664 and 661 nM, respectively (cisplatin was used as a positive control with IC_50_ values of 1.67 and 12.63 μM, respectively) [14]. Compound **24** may be used as an anti-ovarian cancer agent, even in patients who are resistant to platinum compounds. Plausible hypotheses for the biosyntheses of ETPs have been previously reviewed [15].

**Figure 3:**
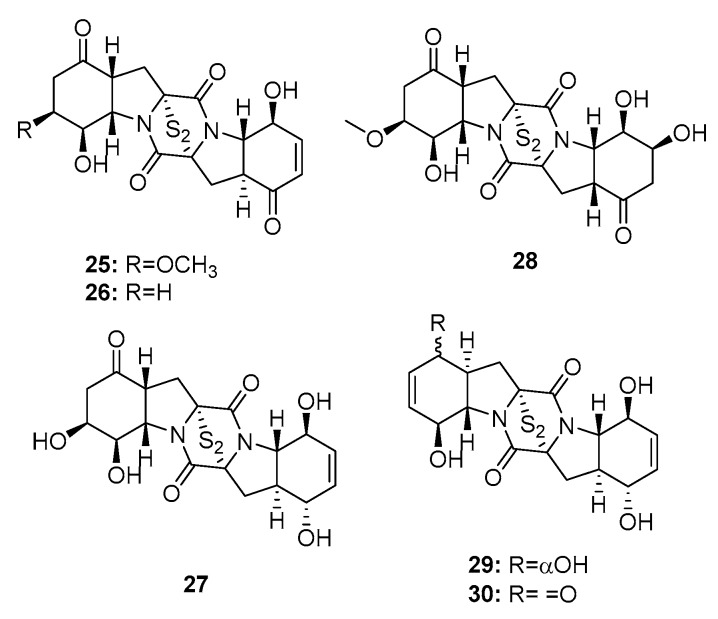
Chemical structures of compounds **25**–**30**.

Four new cytotoxic bisthiodiketopiperazines (brocazines A–F) (**25**–**30**) (Figure 3), which share molecular similarities with gliotoxin, were isolated from a fungal strain of *P. brocae* MA-231, collected from the marine mangrove *Avicennia marina* [16]. Their cytotoxicity was investigated in human prostate cancer (DU145), human cervical carcinoma (Hela), human hepatoma (HepG2), human breast carcinoma (MCF-7), human large-cell lung carcinoma (NCI-H460), SGC-790, human pancreatic cancer (SW1990), human colon carcinoma (SW480), and human glioma (U251) cell lines. Brocazines A, B, E, and F (**25**, **26**, **29**, and **30**) exhibited significant cytotoxic effects against most of the cell lines tested with IC_50_ values in the range of 0.89–9 μM (paclitaxel, cisplatin, cefitinib, doxorubicin, and gemcitabine were used as positive controls with IC_50_ values of 1~11 μM). In contrast, brocazines C and D (**27** and **28**), which lack the α, β unsaturated ketone group, had much lower cytotoxicity (IC_50_ > 20 μM), which suggests that the conjugated ketone system is crucial to the cytotoxic properties of bisthiodiketopiperazine analogues.

**Figure 4:**
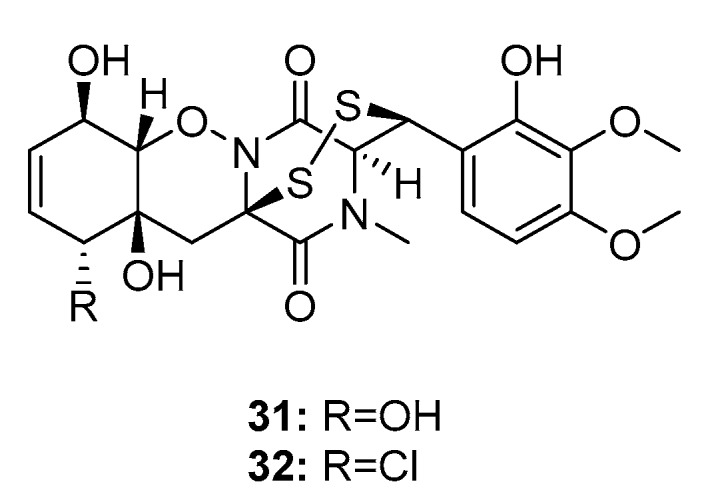
Chemical structures of compounds **31**–**32**.

Two bisthiodiketopiperazines, pretrichodermamide C (**31**) and *N*-methylpretrichodermamide B (**32**) (also called adametizine B and A, respectively) (Figure 4), were isolated from a marine sponge-derived fungus (*P. adametzioides* AS-53) [17], a hyper saline lake-derived *Penicillium* sp. [18], and a marine algicolous fungus (*Penicillium* sp. KMM4672) [19]. All three studies showed that compound **32**, which contains chlorine, exhibited significant cytotoxicity, wherein it reduced the viability of L5178Y mouse lymphoma cells, human prostate cancer 22Rv1 cells, PC-3 cells, LNCaP cells, and brine shrimps (IC_50_ = 2, 0.51, 5.11, 1.76, and 4.8 μM, respectively; while kahalalide F, docetaxel, and colchicine were employed as positive controls with IC_50_ values of 4.3, 0.013, 0.015, 0.004, and 8.1 μM, respectively). Furthermore, it was found active in hormone-resistant 22Rv1 cells at nanomolar concentrations. In contrast, metabolite **31** was completely inactive in all bioassays with IC_50_ values > 100 μM. This remarkable difference in activity indicates that the halogen atom might improve the activity of the metabolite.

**Figure 5:**
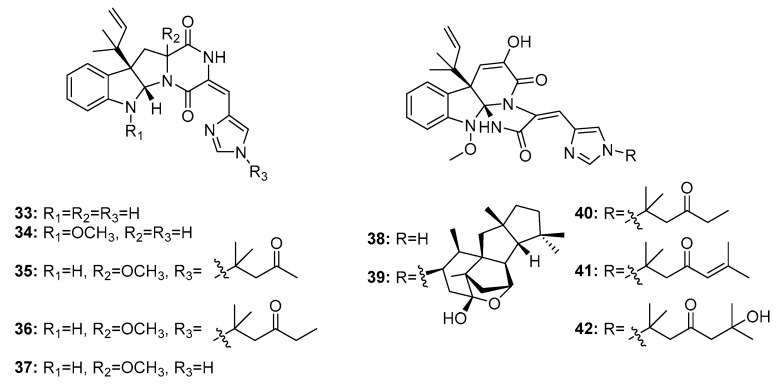
Chemical structures of compounds **33**–**42**.

Roquefortine C (**33**) (Figure 5) is a potential neurotoxin that can activate P-glycoprotein and simultaneously inhibit P450-3A and other hemoproteins [20]. Roquefortine and meleagrin (**38**) analogues are considered biogenetically interrelated mycotoxins with promising cytotoxicity [21]. Recently, a series of roquefortine derivatives, roquefortines F–I (**34**–**37**), and meleagrin analogues, meleagrins B–E (**39**–**42**), were isolated from the deep ocean sediment-derived fungus *Penicillium* sp. [22], and most of these compounds (**34**, **35**, and **39**–**42**) were active against A549, HL-60 (human promyelocytic leukemia), BEL-7402 (human hepatoma), and MOLT-4 (human acute T lymphoblastic leukemia) cancer cell lines. Meleagrin B (**39**) was the most cytotoxic against these four cell lines with IC_50_ values in the range of 1.5–7 μM; the other compounds had IC_50_ values in the range of 4–50 μM. Meleagrin (**38**) was also isolated from a deep sea sediment-derived fungus, *P. commune* SD-118, and was found to be cytotoxic in HepG2, NCI-H460, Hela, MDA-MB-231 (human breast cancer cells), and DU145 human cancer cell lines (IC_50_ = 12, 22, 20, 11, and 5 μg/mL, respectively; while fluorouracil was employed as a positive control with IC_50_ values of 14, 1, 14, 8, and 0.4 μg/mL, respectively) [23].

**Figure 6:**
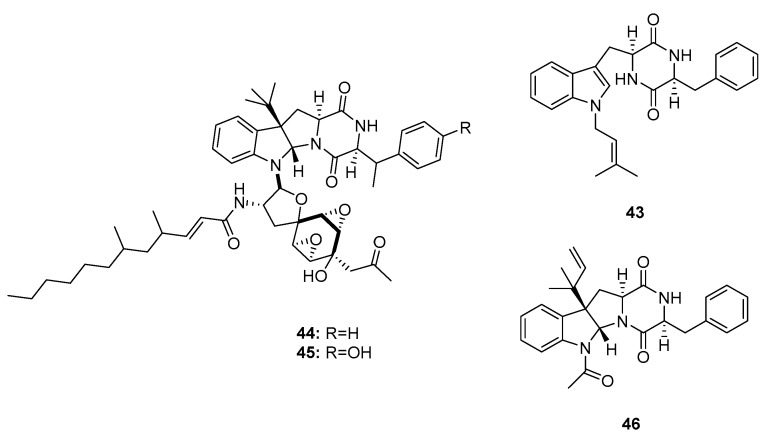
Chemical structures of compounds **44**–**46**.

Penicimutanins A,B (**43**–**45**) and fructigenine A (**46**) (Figure 6) are structurally similar to roquefortines, and were first isolated from diethyl sulfate- or gentamicin-induced mutants of the marine-derived fungus *P. purpurogenum* G59 [24,25]. Mutation-based approaches can activate silent fungal gene clusters and afford more potent metabolites with unique structures. Compounds **44** and **45** are mutant cytotoxic products that showed potent activities against five human cancer cell lines: K562 (human chronic myelogenous leukemia), HL-60, Hela, BGC-823 (human gastric adenocarcinoma), and MCF-7 (IC_50_ values were 5–11 μM for **44** and 8–20 μM for **45**). Compounds **43** and **46** also inhibited the proliferation of these cell lines (Inhibition Rate (IR)% = 22.6 and 20.8 (K562); 17.9 and 55.3 (HeLa); and 26.5 and 65.6% (MCF-7) at 100 μg/mL, respectively; while 5-fluorouracil was employed as a positive control with IR% of 48.5, 37.4, and 47.4 μg/mL at 100 μg/mL, respectively).

**Figure 7:**
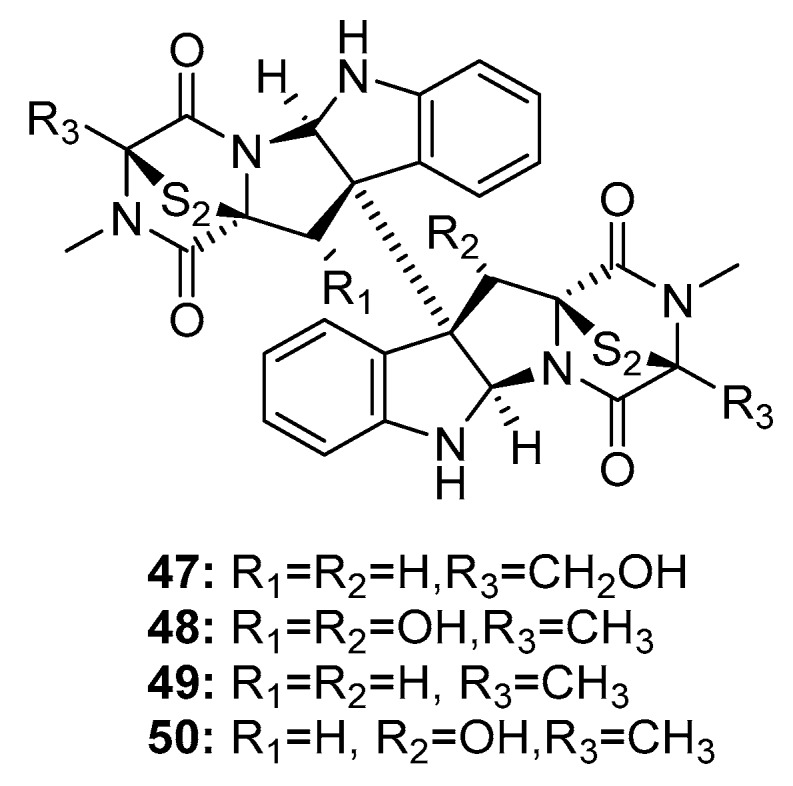
Chemical structures of compounds **47**–**50**.

Since the isolation of (+)-chaetocin A (**47**) and (+)-verticillin A (**48**) (Figure 7) in 1970, dimeric epidithiodiketopiperazine alkaloids have received much attention owing to their diverse biological activities and complex molecular structures [26,27]. In 1999, two additional dimeric epidithiodiketopiperazine alkaloids, (+)-11,11′-dideoxyverticillin A (**49**) and (+)-11′-deoxyverticillin A (**50**), were isolated from a marine alga-derived fungus *Penicillium* sp. and were found to exhibit extraordinary cytotoxicity against HCT-116 cells (human colon cancer) with IC_50_ of 30 ng/mL [28]. Chaetocin A (**47**) was the first compound reported to inhibit HMT, and to have specific effects on HMT SU(VAR)3-9 in vitro and in vivo [29]. (+)-11,11′-Dideoxyverticillin A (**49**), an alkaloid, exhibited diverse antitumor activities in vitro and in vivo [30]; in addition, it potently inhibited the phosphorylation of epidermal growth factor receptor in human breast cancer (MDA-MB-468) [31]. Movassaghi et al. used a concise enantioselective method for the total synthesis of (+)-11,11′-dideoxyverticillin A (**49**) in 2009 [32] based on mimicking the biosynthetic pathway; in addition, they used this approach to synthesize various dimeric epidithiodiketopiperazines [33].

**Figure 8:**
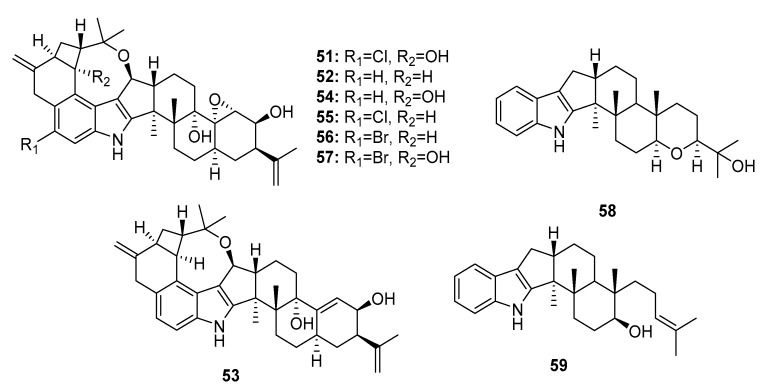
Chemical structures of compounds **51**–**59**.

Seven cytotoxic indole diterpene alkaloids, penitrems A,B (**51**–**52**), D–F (**53**–**55**), paspaline (**58**), and emindole SB (**59**) (Figure 8) were isolated from a marine *Penicillium* sp. KBr-induced mutation of this fungus produced two bromo-substituted indole alkaloids, 6-bromopenitrems B and E (**56**–**57**) [34]. Compounds (**51**–**59**) showed potent antiproliferative (IC_50_ = 5–20 μM for MCF-7; 8–30 μM for MDA-MB-231), anti-migratory (IC_50_ = 7–35 μM for MDA-MB-231) and anti-invasive properties (IR% = 10–75% at 15 μM) against human breast cancer cells. In addition, penitrems A, B, and E (**51**–**52**, **54**) were evaluated in 60 human tumor cell lines as a part of the Development Therapeutics Program of the National Cancer Institute (NCI60). Penitrem B (**52**) exhibited the strongest mean growth inhibitory effect in the 60 human cancer cells (IR% = 41.05% at 10 μM) and was considered a potential selective inhibitory agent for leukemia cells. The nematode *Caenorhabditis elegans* was used to assess the brain’s Maxi-K (BK) channel inhibitory activity and toxicity in vivo [35,36]. Penitrem A (**51**) and 6-bromopenitrem E (**57**) displayed BK channel inhibition, comparable to that of a knockout strain. A pharmacophore study on the effects of the penitrem skeleton on the antiproliferative activity against MCF-7 cells indicated that less structural complexity of the penitrems, paspaline (**58**), and emindole SB (**59**) better maintained the molecular alignment and pharmacophoric features. Penitrem A (**51**) was also considered a neurotoxin that antagonizes BK channels [37].

**Figure 9:**
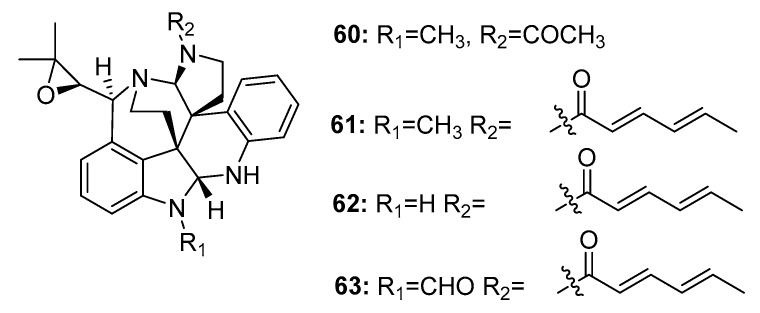
Chemical structures of compounds **60**–**63**.

Another large family of indole alkaloid mycotoxins, comprising communesins A–D (**60**–**63**) (Figure 9), was isolated from marine-derived *Penicillium* sp. from a marine alga [38], marine sponge [39], and marine sediment [40]. Communesin B (**61**) (also called nomofungin) was more cytotoxic to P388 lymphocytic leukemia cells (ED_50_ = 0.45 μg/mL) than communesin A (**60**) (ED_50_ = 3.5 μg/mL). The antiproliferative activity of communesins B–D (**61**–**63**) was further evaluated in six lymphocytic leukemia cell lines (U-937, THP-1, NAMALWA, L-428, MOLT-3, and SUP-B15). They steadily and effectively inhibited the proliferation of five of these cell lines with ED_50_ values ranging from 7 to 16 μg/mL; however, they were inactive in L-428 cells. The total synthesis of communesin B (**61**) was previously reported [41].

**Figure 10:**
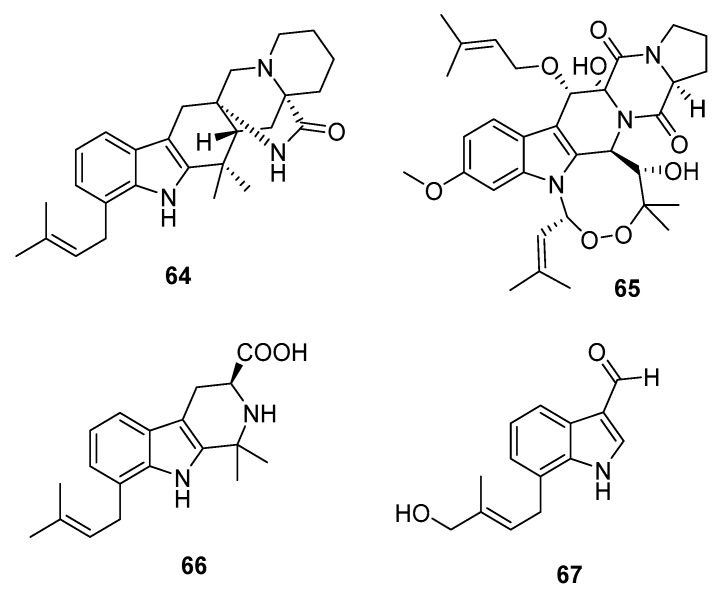
Chemical structures of compounds **64**–**67**.

Four new cytotoxic prenylated indole alkaloid derivatives, penioxamide (**64**) [42], 13-*O*-prenyl-26-hydroxyverruculogen (**65**) [43], and penipalines B and C (**66**–**67**) (Figure 10) [44], were isolated from marine mangrove-derived *P. oxalicum* EN-201, marine sediment-derived *P. brefeldianum* SD-273, and marine sediment-derived *P. paneum* SD-44, respectively. Metabolites **64**–**65** showed significant lethality in brine shrimps with LD_50_ values of 5.6 and 9.4 μM, respectively (colchicine was employed as a positive control with an LD_50_ value of 7.8 μM). Metabolites **66**–**67** induced moderate cytotoxicity against A549 (IC_50_ = 20.44 and 21.54 μM, respectively) and HCT-116 cell lines (IC_50_ = 14.88 and 18.54 μM, respectively).

**Figure 11:**
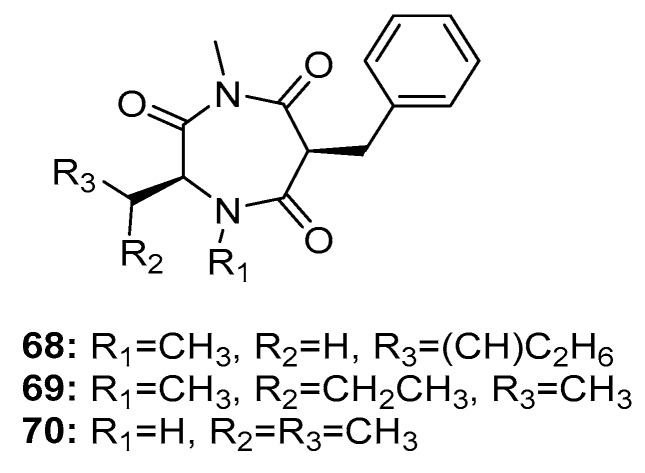
Chemical structures of compounds **68**–**70**.

In addition, three 1,4-diazepane derivatives, terretriones A, C, and D (**68**–**70**) (Figure 11), obtained from marine sponge-derived *P. vinaceum* [45] and marine tunicate-derived *Penicillium* sp. CYE-87 [46], moderately inhibited the migratory activity of MDA-MB-231 cells with IC_50_ values of 17.7, 17.6, and 16.5 μM, respectively (Z-4-ethylthio-phenylmethylene hydantoin was used as a positive control with an IC_50_ value of 43.4 μM). These findings indicate that terretriones might be potential anti-metastatic breast cancer candidates.

**Figure 12:**
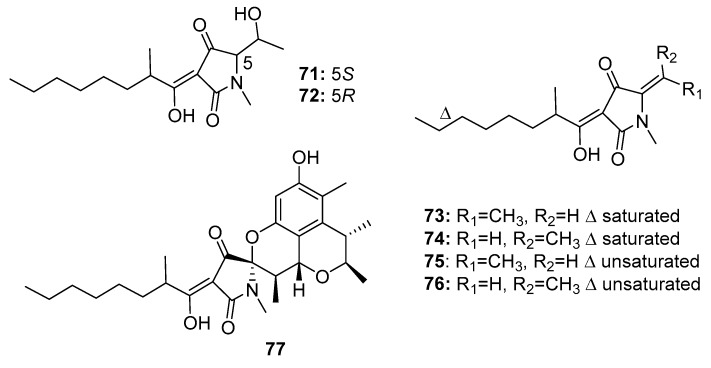
Chemical structures of compounds **71**–**77**.

Six tetramic acid derivatives, penicillenols A1, A2, B1, B2, D1, and D2 (**71**–**76**) (Figure 12), were isolated from a marine sediment-derived fungus *P. citrinum*. Penicillenol B2 (**74**) exhibited the strongest cytotoxic activity against A-375 human malignant melanoma cell line (IC_50_ = 0.97 μg/mL), whereas the IC_50_ values of compounds **71**–**73** were 3.2, 13.8, and 2.8 μg/mL, respectively [47,48]. Penicillenols D1 and D2 (**75**–**76**) showed moderate cytotoxicity against A549 cells with IC_50_ values of 17.2 and 12.1 μg/mL, respectively. However, penicillenols A1 and B1 (**71**, **73**) showed significant cytotoxicity in HL-60 cells (IC_50_ = 0.76 and 3.2 μM, respectively) [49]. A novel tetramic acid derivative, penicitrinine A (**77**), which contains a citrinin-like group, was isolated [50]. The combination of two cytotoxic fragments in this metabolite might contribute to its extensive antiproliferative activity in diverse tumor cell lines, particularly A-375 cells. Penicitrinine A (**77**) not only induced A-375 cell apoptosis by upregulating Bax and downregulating Bcl-2, but also inhibited A-375 cell metastatic activity by suppressing matrix metalloproteinase 9 (MMP-9) and promoting the expression of its specific inhibitor, tissue inhibitor of metalloproteinases-1 (TIMP-1). These findings suggest that penicitrinine A (**77**) is a potential lead compound.

**Figure 13:**
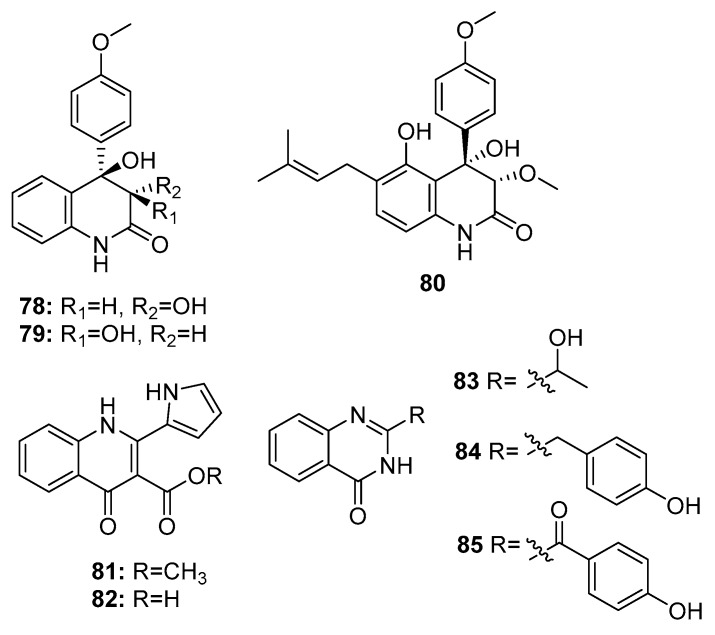
Chemical structures of compounds **78**–**85**.

Quinolinone and quinazolinone alkaloids have unique pharmacophores that allow their binding to multiple sites with high affinity; moreover, they possess various biological properties [51]. Some cytotoxic quinolinone (**78**–**82**) and quinazolinone alkaloids (**83**–**85**) (Figure 13) were isolated from marine-derived members of the *Penicillium* genus, such as *P. janczewskii*, *Penicillium* sp. ghq208, *P. oxalicum* 0312F1, *P. chrysogenum* EN-118, and *P. commune* SD-118 [23]. 2-quinolinone metabolites (**78**–**79**) exhibited IR% values of 50–60% at 10 μg/mL. Interestingly, compound **80**, which has an additional prenyl chain, showed significant cytotoxicity against MDA-MB-231 and HT-29 (human colon carcinoma) cell lines with IR% values of 92–96% at 10 μg/mL [52]. In addition, a 4-quinolinone derivative (**82**) exhibited significant cytotoxicity against the human lung cancer cell line 95-D (IC_50_ = 0.57 μg/mL). Both compounds **81** and **82** exhibited similar cytotoxicity (IC_50_ = 11.3 and 13.2 μM, respectively) against HepG2 cells [53,54]. However quinazolinone derivatives (**83**–**85**) showed only moderate cytotoxicity (compound **83**, IC_50_ = 20 μg/mL in SW1990 cell line; compound **84**, IC_50_ = 8 μg/mL in DU145, A549, and Hela cell lines; and compound **85**, IR% = 35–40 at 200 μg/mL in SGC-7901 and BEL-7404 cell lines) [55,56].

**Figure 14:**
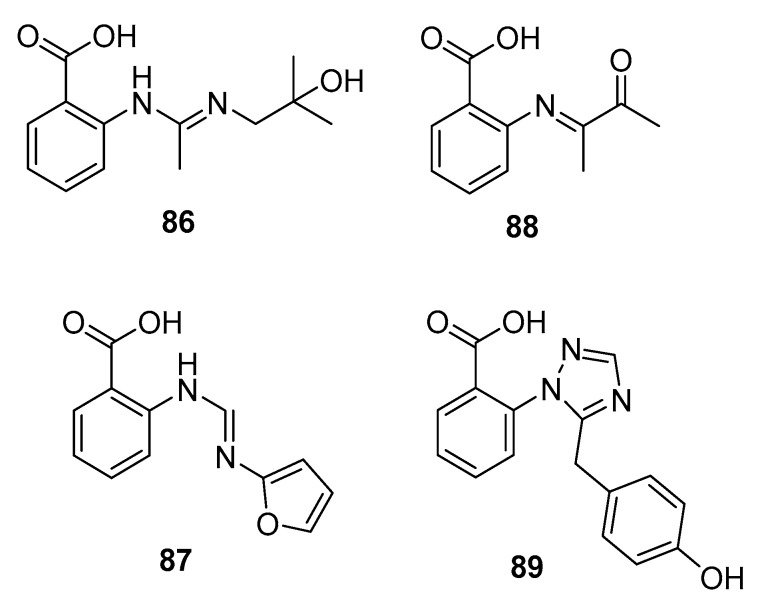
Chemical structures of compounds **86**–**89**.

In an ongoing study that aims to produce new active metabolites from *P. paneum* SD-44 (a deep sea sediment-derived fungus) using culture variations, three amidine anthranilic acid analogues (**86**–**88**) and one triazole anthranilic acid derivative, penipanoid A (**89**) (Figure 14), were obtained after culture in malt and rice medium, respectively. Compounds **86** and **87** strongly inhibited RKO human colon cancer cell viability (IC_50_ = 8.4 and 9.7 μM, respectively). In addition, compound **88** was cytotoxic to Hela cells (IC_50_ = 6.6 μM) [57], whereas compound **89** with a triazole group only weakly inhibited SMMC-7721 cell viability (human hepatocarcinoma) (IC_50_ = 54.2 μM) while fluorouracil was used as a positive control for three cell lines with IC_50_ values of 25.0, 14.5, and 13.0 μM, respectively [58].

**Figure 15:**
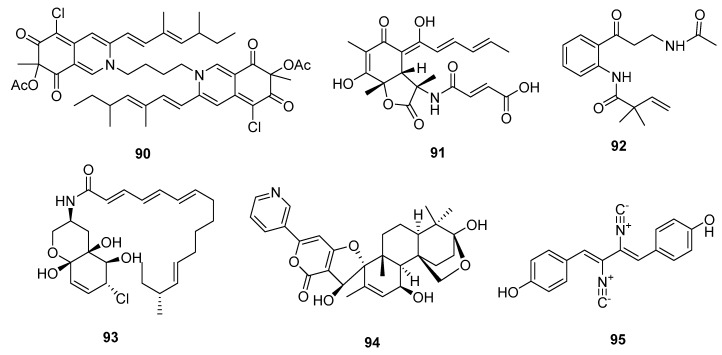
Chemical structures of compounds **90**–**95**.

An azaphilone analogue, bis-sclerotioramin (**90**) (Figure 15), obtained from a marine mangrove endophytic fungus, *Penicillium* 303#, was found to possess moderate cytotoxicity against MDA-MB-435 cell line (IC_50_ = 7.13 μg/mL), while epirubicin was used as a positive control with an IC_50_ value of 0.325 μg/mL [59]. Another novel alkaloid, the sorbicilin-derived sorbicillactone A (**91**), was first isolated from a Mediterranean sponge-derived fungus, *P. chrysogenum*. Sorbicillactone A (**91**) exhibited a selective antileukemic activity in L5178Y cells (murine leukemic lymphoblast) with an IC_50_ of 2.2 μg/mL, as well as in other tumor cell lines (IC_50_ > 10 μg/mL). The biosynthesis of sorbicillactone A (**91**) was investigated using ^13^C-labeled precursor feeding experiments, which showed that the its skeleton was derived from acetate, alanine, and methionine [60]. Furthermore, a new strategy for the large-scale biotechnological production of sorbicilin-derived alkaloids was developed for preclinical screening and a structure-activity relationship (SAR) study [61]. In addition, a 4-oxoquinoline derivative, brocaeloid B (**92**), isolated from the mangrove endophytic fungus *P. brocae*, showed mild lethality against brine shrimps with an LD_50_ of 36.7 μM, while colchicine was used as a positive control with an LD_50_ value of 87.6 μM [62]. Li et al. cultured the marine mangrove fungus *P. varibile* with the DNA methyltransferase inhibitor 5-azacytidine to identify novel responsive molecules by gene silencing. A highly modified fatty acid amide, varitatin A (**93**), exhibited significant cytotoxicity against HCT-116 cells (IC_50_ = 2.8 μM, while doxorubicin was used as a positive control with an IC_50_ value of 0.2 μM) and potently inhibited protein tyrosine kinases, platelet-derived growth factor receptor-beta (PDGFR-β), and ErbB4 with IR% values of 50 and 40%, respectively, at a concentration of 1 μM [63]. In addition, a new pyridinyl-α-pyrone alkaloid, 18-hydroxydecaturin B (**94**), was isolated from an endophytic fungus, *P. oxalicum* EN-201, derived from the marine mangrove *Rhizophora stylosa*. Compound **94** showed significant lethality in brine shrimps (LD_50_ = 2.3 μM, while colchicine was used as a positive control with an LD_50_ value of 7.8 μM) [42]. A previous study showed that the metabolites of decaturin, a potent insecticide, were cytotoxic [64]. The isocyanide alkaloid, xantocillin X (**95**), which is a known antiviral and antibiotic agent [65], was first isolated from *P. notatum* in 1950 [66]. Recently, compound **95** was isolated from the deep sea sediment-derived fungus *P. commune* SD-118, and showed moderate cytotoxicity in six cancer cell lines (MCF-7, HepG2, NCI-H460, Hela, DU145, and MDA-MB-231) with IC_50_ values of 12, 7, 10, 10, 8, and 8 μg/mL, respectively, while fluorouracil was used as a positive control with IC_50_ values of 4, 14, 1, 14, 0.4, and 8 μg/mL, respectively [23]. A later pharmacological study on human HepG2 cells showed that compound **95** induced apoptosis and autophagy by inhibiting the MEK/EPK signaling pathway and activating the class III PI3K/Beclin 1 signaling pathway [67].

## 3. Terpenes, Meroterpenes, and Steroids

**Figure 16:**
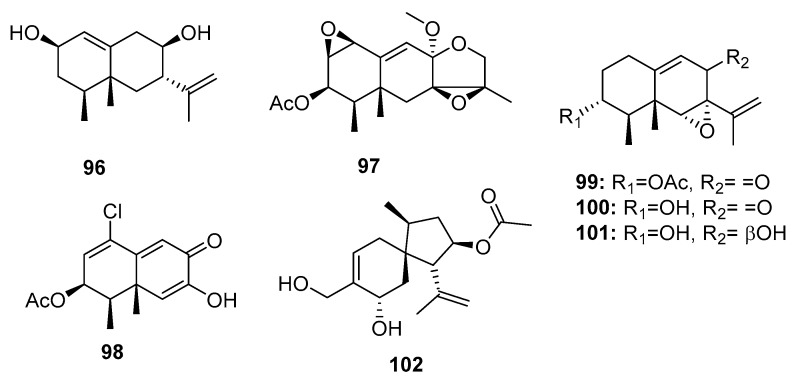
Chemical structures of compounds **96**–**102**.

The genus *Penicillium* is a well-known producer of eremophilane-type sesquiterpenes with phytotoxic, mycotoxic, and phytohormonic activities [68,69]. Chemical investigation of an Antarctic deep sea-derived fungus, *Penicillium* sp. PR19 N-1, yielded three new cytotoxic eremophilane-type sesquiterpenes (**96**–**98**) (Figure 16), which were moderately cytotoxic to HL-60 (IC_50_ = 45.8, 28.3, and 11.8 μM, respectively) and A549 (IC_50_ = 82.8, 5.2, and 12.2 μM, respectively) cancer cell lines [70,71]. Three other eremophilane-type sesquiterpenes (**99**–**101**) were isolated from a sea mud-derived fungus, *Penicillium* sp. BL 27-2. Of these, compound **99** was the most cytotoxic to P388, A549, HL-60, and BEL-7402 cell lines (IC_50_ = 0.073, 0.096, 0.065, and 4.59 μM, respectively), whereas compounds **100** and **101** had IC_50_ values in the range of 3–12 μM [72]. These results suggest that the epoxide ring is essential for the cytotoxicity of eremophilane-type sesquiterpenes and that the presence of an acetyl group enhances the cytotoxicity. A new acorane sesquiterpene, adametacorenol B (**102**), isolated from a marine sponge-derived fungus, *P. adametzioides* AS-53, displayed selective cytotoxicity against NCI-H446 cell lines (IC_50_ = 5 μM), compared to its cytotoxicity against the other 13 tumor cell lines tested (A549, DU145, HeLa, HepG2, Huh-7 (human hepatocarcinoma), L02 (human hepatocarcinoma), LM3 (murine breast cancer), MA (mouse Leydig tumor), MCF-7, SGC-7901, SW1990, SW480, and U251) (IC_50_ > 10 μM) [17].

**Figure 17:**
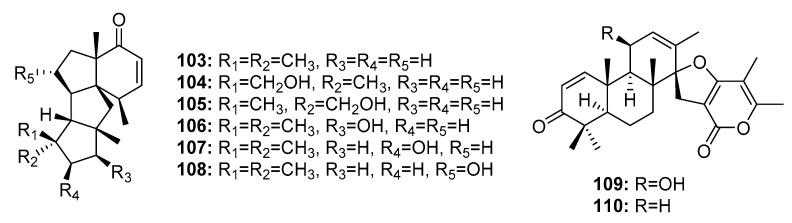
Chemical structures of compounds **103**–**110**.

The deep sea sediment-derived fungus *Penicillium* sp. was reported to be a good source of cytotoxic diterpenes. Six tetracyclic diterpenes, conidiogenones B–G (**103**–**108**) (Figure 17), exhibited cytotoxicity against HL-60, A549, BEL-7402, and MOLT-4 cell lines. Conidiogenone C (**104**) was potently cytotoxic against HL-60 and BEL-7402 cells with IC_50_ values of 0.038 and 0.97 μM, respectively; however, it was not cytotoxic against A549 and MOLT-4 cell lines at 50 μM. Other conidiogenones (**103**, **105**–**108**) had moderate cytotoxicity with IC_50_ values ranging from 1 to 50 μM [22]. The spiroditerpenes, breviones I and A (**109**–**110**) were also obtained from this fungus and showed cytotoxicity comparable to that of cisplatin (the positive control) against MCF-7 cells (IC_50_ = 7.44 and 28.4 μM, respectively, versus 8.04 μM for cisplatin) [73].

**Figure 18:**
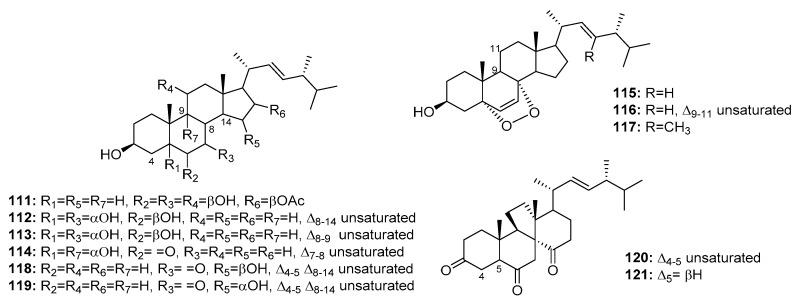
Chemical structures of compounds **111**–**121**.

Although several marine-derived steroids have been isolated, few have been found to be bioactive. A cytotoxic polyoxygenated steroid, penicisteroide A (**111**) (Figure 18), was isolated from a marine alga-derived fungus, *P. chrysogenum* QEN-24S. Penicisteroide A (**111**) displayed moderate cytotoxicity against Hela, SW1990, and NCI-H460 cell lines with IC_50_ values of 15, 31, and 40 μg/mL, respectively [74]. Three other polyoxygenated steroids (**112**–**114**) and two epidioxygenated steroids (**115**–**116**) were isolated from the marine moss-derived fungus *Penicillium* sp. These steroids moderately inhibited HepG2 cell line growth (IC_50_ values = 10.4, 15.6, 20.7, 16.8, and 21.3 μg/mL, respectively) [75]. In addition, an epidioxygenated steroid (**117**), produced by a sea squirt-derived fungus, *P. stoloniferum* QY2-10, was cytotoxic to P388 cells with an IC_50_ of 4.07 μM [76]. Moreover, a marine *Penicillium* sp. fungus collected from the inner tissues of an unidentified sponge is reportedly the source of two epimeric steroids (**118**–**119**) and two cytotoxic steroids of a new class, dankasterone A (**120**) and B (**121**). Dankasterone A (**120**) was more effective than the positive control, adriamycin (IC_50_ = 0.98 μM) against HL-60, Hela, and K562 cancer cell lines with IC_50_ values of 0.78, 4.11, and 7.57 μM, respectively. Compounds **118**–**119** and **121** also significantly inhibited K562 cell growth (IC_50_ = 4.38, 5.54, and 7.89 μM, respectively) [77].

**Figure 19:**
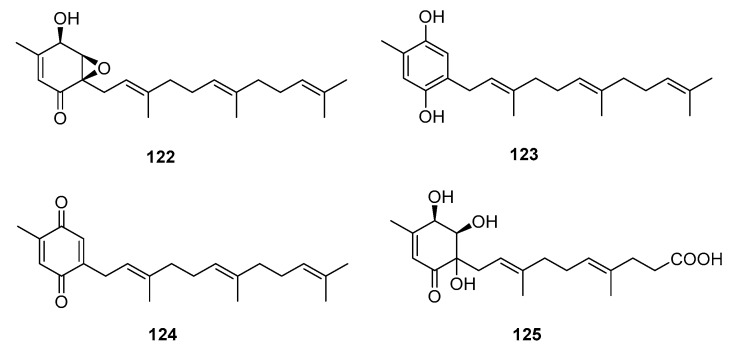
Chemical structures of compounds **122**–**125**.

Meroterpenes are widely distributed in the marine environment, particularly in brown algae and microorganisms. Terpene-quinone and -hydroquinone are the major bioactive members because they produce reactive oxygen species (ROS) [78]. Three quinone- and hydroquinone-type meroterpenes (**122**–**124**) (Figure 19) were isolated from a marine-derived *Penicillium* sp. Compounds **122** and **123** exhibited extensive cytotoxicity against five cancer cell lines (A549, SKOV-3 (human ovary adenocarcinoma), SKMEL-2 (human skin cancer), XF498 (human CNS cancer), and HCT15 (human colon cancer)) with IC_50_ values in the range of 3–10 μg/mL, whereas compound **124** had IC_50_ values ranging from 20 to 40 μg/mL (doxorubicin was used as a positive control with IC_50_ values of 0.02~0.8 μg/mL). These results suggest that the quinone form tends to be less cytotoxic [79]. Penicillone A (**125**), isolated from marine-derived *Penicillium* sp. F11., contains a carboxylic acid group instead of the isoprenyl tail, which resulted in mild cytotoxicity against fibrosarcoma (HT1080) and human nasopharyngeal carcinoma (Cne2) cell lines (IC_50_ = 45.8 and 46.2 μM, respectively) [80].

**Figure 20:**
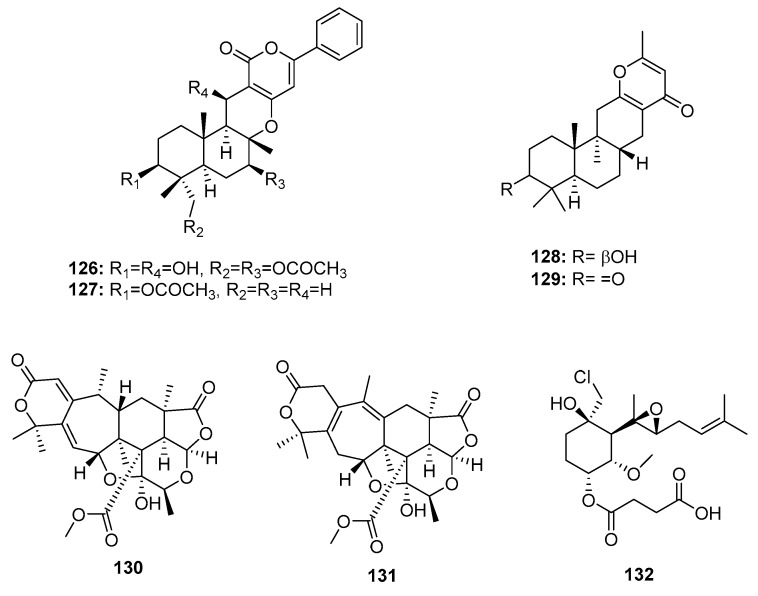
Chemical structures of compounds **126**–**132**.

Two sesquiterpene α-pyrones, phenylpyropenes E and F (**126**–**127**) (Figure 20), were isolated from the marine-derived fungus *P. concentricum* ZLQ-69 and displayed moderate and selective cytotoxicity against MGC-803 cells (human gastric cancer) with IC_50_ values of 19.1 and 13.6 μM, respectively (doxorubicin was used as a positive control with an IC_50_ value of 0.37 μM) [81]. Furthermore, the marine sediment-derived fungus *Penicillium* sp. F446 yielded two new sesquiterpene γ-pyrone-type meroterpenes, penicillipyrone A and B (**128**–**129**), which were moderately cytotoxic against A549 cells (IC_50_ = 15 and 17 μM, respectively, while doxorubicin was used as a positive control with an IC_50_ value of 1.2 μM) [82]. Two polycyclic α-pyrone-type meroterpenes (**130**–**131**), isolated from the marine mangrove endophytic fungus *Penicillium* 303#, exhibited IC_50_ values of 20–30 μg/mL in four cancer cell lines (MDA-MB-435, HepG2, HCT-116, and A549), while epirubicin was used as a positive control with IC_50_ values of 0.2~0.6 μg/mL [59]. Fumagillin was first isolated from *Aspergillus fumigatus* in 1949, and has been used as an antimicrobial [83]. Recently, ligerin (**132**), a natural chlorinated merosesquiterpene related to fumagillin, was obtained from a marine-derived *Penicillium* sp., and showed selective in vitro antiproliferative activity against osteosarcoma cell lines (IC_50_ = 117 nM against POS1 cells, which is 20 times greater than the IC_50_ in other cancer cell lines), while doxorubicin was used as a positive control with IC_50_ values of 0.04~2 μM [84]. Ligerin analogues were semi-synthesized in an SAR study, which showed that chlorohydrin and C6 substituents were crucial for cytotoxic activities. Furthermore, ligerin (**132**) exhibited stronger cytotoxicity against human osteosarcoma SaOS2 and MG63 cancer cell lines. However, its cytotoxicity was less than that of TNP470 (a positive control and fumagillin analogue) [85].

## 4. Polyketides

**Figure 21:**
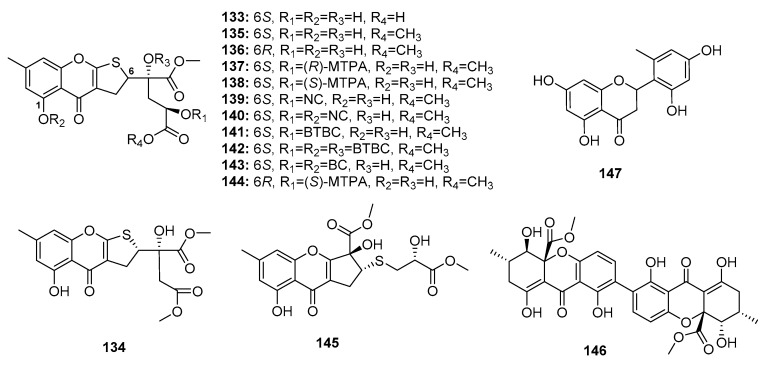
Chemical structures of compounds **133**–**147**.

Chromone derivatives are abundantly present in nature and are considered potential immunomodulatory, anticancer, and anti-inflammatory agents. Chromone scaffolds were reported to possess outstanding pharmacological properties [86]. A Chinese research group recently isolated four dihydrothiophene-condensed chromones, oxalicumones D, E (**133**–**134**) and A, B (**135**–**136**) (Figure 21) from a marine gorgonian-derived fungus, *P. oxalicum* SCSGAF 0023. Similar to synthetic dihydrothiophene-condensed chromones (**137**–**144**), these four natural chromones (**133**–**136**) displayed significant cytotoxicity against eight carcinoma cell lines (human lung adenocarcinoma (H1975), human lymphoma (U937), K562, BGC823, MOLT-4. MCF-7, HL-60, and Huh-7) (IC_50_ < 10 μM). Of these, oxalicumone A (**135**) was the most cytotoxic against MOLT-4 cell line (IC_50_ = 0.30 μM). An SAR study showed that the 2,3-dihydrothiophene unit was crucial for activity and that the presence of 1-OH and absolute configuration at C-6 contributed to cytotoxicity [87,88]. Subsequent pharmacological studies showed that oxalicumone A (**135**) inhibited leukemia cell growth and induced apoptosis, in part, via the induction of the endoplasmic reticulum stress pathway by upregulating calnexin and Bax and activating unfolded protein response [89]. Another study found that oxalicumone A (**135**) could induce oxidative stress injury in the mitochondria, and thus promote human renal epithelial cell death [90]. Chromosulfine (**145**), a novel cyclopentachromone sulfide which is structurally similar to dihydrothiophene-condensed chromones, was isolated from a neomycin-resistant mutant of the marine-derived fungus *P. purpurogenum* G59, and showed selective cytotoxicity against HL-60 cancer cell line (IC_50_ = 16.7 μM) [91]. Secalonic acid F (**146**), a chiral dimeric tetrahydroxanthone, was first isolated from *Aspergillus* sp. before discovering that the deep sea sediment-derived fungus *Penicillium* sp. F11 is a good source of this compound. Compound **146** induced HL-60 cell apoptosis by modulating the Rho GDP dissociation inhibitor 2 pathway [92]. Recent studies showed that secalonic acid F (**146**) could induce apoptosis by activating caspase 3 and 9 through the mitochondrial pathway in hepatocellular carcinoma, wherein it was found to be more effective than 5-fluorouracil [93]. Furthermore, a flavone, namely penimethavone A (**147**), obtained from a gorgonian-derived fungus, *P. chrysogenum*, exhibited selective cytotoxicity against Hela and rhabdomyosarcoma cell lines (IC_50_ = 8.41 and 8.18 μM, respectively) while adriamycin was used as a positive control with IC_50_ values of 0.43 and 0.09 μM, respectively [94].

**Figure 22:**
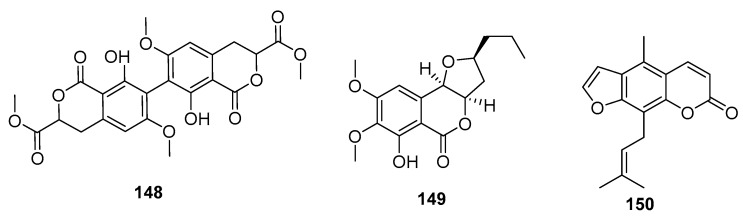
Chemical structures of compounds **148**–**150**.

Coumarin derivatives of the chromone isomers (**148**–**150**) (Figure 22) were also isolated from the deep sea sediment-derived fungus (*P. chrysogenum* SCSIO 41001), a marine sponge-derived fungus *Penicillium* sp., and a mangrove endophytic fungus (*Penicillium* sp. ZH16), respectively. The dimeric isocoumarin, bipenicillisorin (**148**), displayed significant cytotoxicity against K562, A549, and Huh-7 cell lines (IC_50_ = 6.78, 6.94, and 2.59 μM, respectively), while taxol was used as a positive control with IC_50_ values of 3.44, 2.61, and 14.70 nM, respectively [95]. The dihydroisocoumarin monocerin (**149**) exhibited significant cytotoxicity against L5178Y cells (a murine lymphoma cell line) with an IC_50_ value of 8.4 μM (kahalalide F was used as a positive control with an IC_50_ value of 4.3 μM) [96]. Moreover, furanocoumarin (**150**) showed moderate cytotoxicity against human nasopharyngeal carcinoma (KB and KBv200) cell lines (IC_50_ = 5 and 10 μg/mL, respectively) [97].

**Figure 23:**
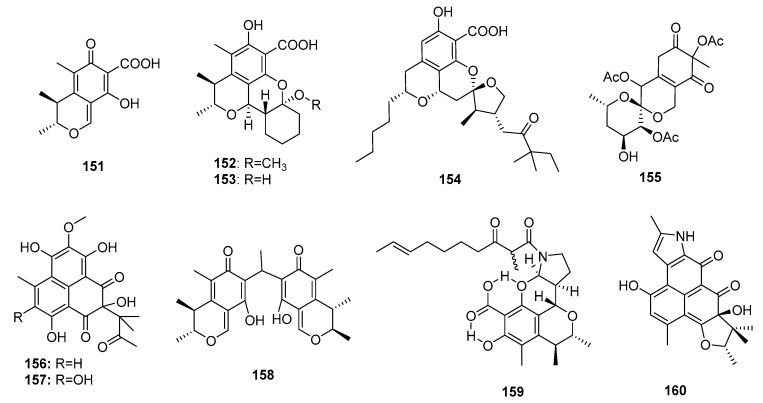
Chemical structures of compounds **151**–**160**.

Citrinin (**151**) (Figure 23), a typical azaphilone polyketide mycotoxin, was first found in *P. citrinum* in 1931 [98]. Compound **151** is strongly nephrotoxic because of its inhibition of respiration complex III [99]. The biosynthesis pathway of compound **151** was further investigated [100]. Interestingly, the marine sponge-derived fungus *Penicillium* sp. FF001 was found to be a good source of unique and potent citrinin derivatives [101]. Two new citrinin derivatives, penicitrinols L and M (**152**–**153**), isolated from the marine sediment-derived fungus *P. citrinum*, showed moderate cytotoxicity against a human Caucasian colon adenocarcinoma cell line (SW-620) (IC_50_ = 25.6 and 20.9 μM, respectively) [48]. One penicitrinol analogue, berkelic acid (**154**), with a novel spiroketal structure, isolated from an acid mine lake fungal extremophile *Penicillium* sp., showed selective and extraordinary cytotoxicity against a human ovarian carcinoma cell line (OVCAR-3) at nanomolar concentrations (GI_50_ = 91 nm) [102]. The total synthesis of (–)-berkelic acid (**154**) was previously described [103]. An alga-derived fungus, *P. thomii*, yielded a new citrinin analogue, sargassopenilline C (**155**), which possessed a unique 6,6-spiroketal skeleton and inhibited the transcription of oncogenic nuclear factor, AP-1 (IC_50_ = 15 μM) [104]. Two phenalenone-skeleton citrinin analogues, sculezonones A and B (**156**–**157**), isolated from a marine sponge-derived fungus *Penicillium* sp., inhibited both DNA polymerases (α and β) [105]. Dicitrinone B (**158**), a marine sediment-derived fungal metabolite (*P. citrinum*) containing a rare carbon-bridge citrinin dimer, induced A-375 cell apoptosis by generating ROS via a caspase-related pathway [106]. In another study, two novel skeletal metabolites (**159**–**160**) possibly biogenetically derived from citrinin were found. Perinadine A (**159**), a scalusamide A-type pyrrolidine isolated from a fish gastrointestinal fungus, *P. citrinum*, exhibited mild cytotoxicity against a murine leukemia L1210 cell line (IC_50_ = 20 μg/mL) [107]. However, herqueiazole (**160**), obtained from a marine sediment-derived fungus, *Penicillium* sp. F011, possessed a novel pyrrole-containing phenalenone moiety and demonstrated weak cytotoxicity against A549 cells (IC_50_ = 67.3 μM), while doxorubicin was used as a positive control with an IC_50_ value of 3.3 μM [108].

**Figure 24:**
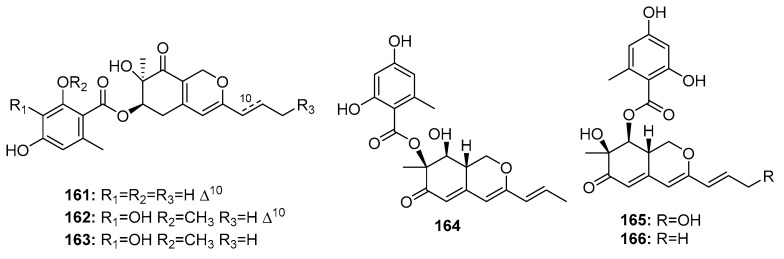
Chemical structures of compounds **161**–**166**.

Other fungal azaphilone polyketides include comazaphilones D–F (**161**–**163**) (Figure 24), pinophilins A, B, and Sch 725680 (**164**–**166**), which were isolated from a marine sediment-derived fungus, *P. commune* QSD-17 (comazaphilones D–F), and a marine seaweed-derived *P. pinophilum* Hedgcok (pinophilins A-B and Sch 725680). Comazaphilones D–F (**161**–**163**) showed selective but weak cytotoxicity against SW1990 cell line (IC_50_ = 51, 26, and 53 μM, respectively), while fluoruoracil was used as a positive control with an IC_50_ value of 120 μM) [109]. Azaphilone derivatives (**164**–**166**) were suggested to suppress cancer cell proliferation by inhibiting DNA replication via the inhibition of mammalian DNA polymerases A, B, and Y [110].

**Figure 25:**
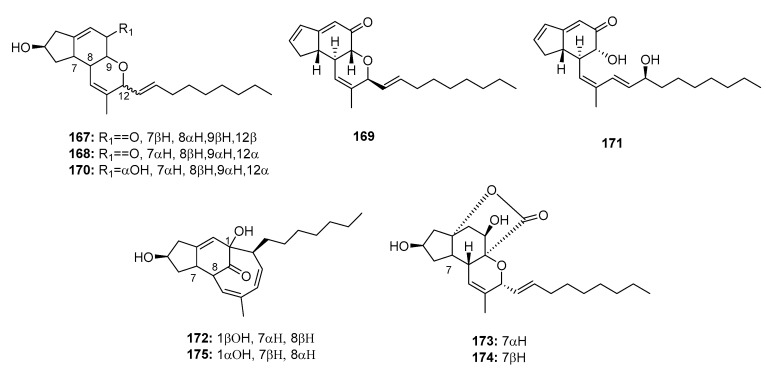
Chemical structures of compounds **167**–**174**.

*Penicillium* sp. strain OUPS-79, which is derived from the marine alga *Enteromorpha intestinalis*, yielded various cytotoxic polyketides, including penostatins A–C, E–I (**167**–**169**, **171**–**175**) (Figure 25) [111,112]. They were found to be significantly cytotoxic to P388 lymphocytic leukemia cells (ED_50_ = 0.8, 1.2, 1.0, 0.9, 1.4, 0.5, 0.8, and 1.2 μg/mL, respectively). However, penostatin D (**170**) exhibited moderate cytotoxicity (ED_50_ = 11.0 μg/mL), which may be attributed to the absence of the cyclic conjugated enone system. Moreover, penostatin C (**169**) exhibited significant cytotoxicity in seven of the 36 cell lines tested with ED_50_ values ranging from 1 to 2 μg/mL. Recent studies have shown that penostatins A–C (**167**–**169**) may be tyrosine phosphatase 1B (PTP1B) inhibitors, which can be used to treat type II diabetes and other associated metabolic diseases (IC_50_ = 15.87, 33.65, and 0.37 μM, respectively), while sodium orthovanadate was used as a positive control with an IC_50_ value of 0.65 μM [113]. The total synthesis of penostatins A, B, and F (**167**, **168**, **172**) was previously reported [114,115].

**Figure 26:**
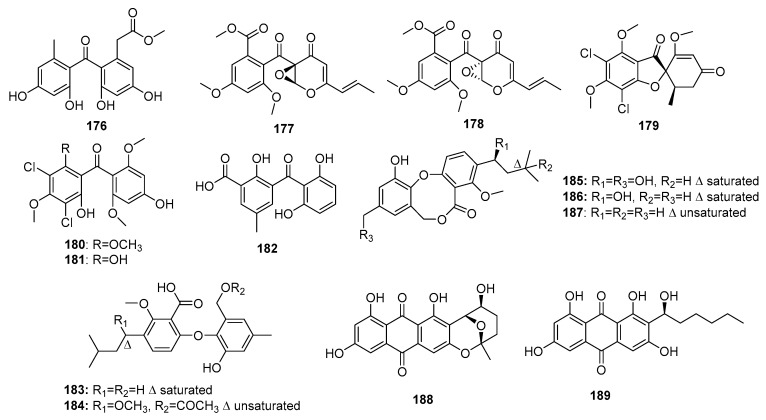
Chemical structures of compounds **176**–**189**.

Fungal phenolic polyketides have diverse biological activities and unique structures [116]. A weak DNA topoisomerase Ι inhibitor, compound (**176**) (Figure 26), was obtained from the marine sediment-derived *P. oxalicum* HSY05 [117], whereas a racemic mixture (**177**–**178**) was obtained from the co-cultivation of marine mangrove-derived *Penicillium* sp. WC-29-5 and *Streptomyces fradiae* 007. Compounds **177**–**178** displayed significant cytotoxicity against H1975 cell lines (IC_50_ = 3.97 and 5.73 μM, respectively). Moreover, compound **178** exhibited cytotoxicity against HL-60 cells (IC_50_ = 3.73 μM) [118]. Using a bioinformatics tool, Marine Halogenated Compound Analysis (MeHaloCoA), three halogenated bioactive metabolites, (+)-5-chlorogriseofulvin (**179**) as well as griseophenones I and G (**180**–**181**), were isolated from a marine-derived *P. canescens*. They inhibited the growth of KB cells at a concentration of 0.6 μM (IR% = 49, 58, and 47%, respectively) [119]. Furthermore, one benzophenone, iso-monodictyphenone (**182**), and two diphenyl ether derivatives, penikellides A and B (**183**–**184**), were isolated from a mangrove endogenous fungus, *Penicillium* sp. MA-37. These three metabolites exhibited moderate brine shrimp lethality (LD_50_ = 25.3, 14.2, and 39.2 μM, respectively), while colchicine was used as a positive control with an LD_50_ value of 1.22 μM [120]. Penicillide (**185**), a multifunctional metabolite produced by a marine sediment-derived *Penicillium* sp. strain, was shown to be an acyl-CoA cholesterol acyltransferase (ACAT) [121], nonpeptide calpain inhibitor [122], and oxytocin antagonist [123]. Furthermore, compound **185** was found to exhibit cytotoxic, antibiotic, and plant growth inhibitory properties. Recently, two marine fungi, *P. pinophilum* (derived from a gorgonian) and *Penicillium* sp. ZLN29 (derived from a sediment), were found to produce penicillide (**185**) and penicillide derivatives (**186**–**187**) that exhibited potent cytotoxicity against HepG2 cell line (IC_50_ = 9.7 and 9.9 μM for **185**–**186**, respectively); moreover, compound **187** showed additional cytotoxicity against Hela cell line (IC_50_ = 6.1 μM) [124,125]. Two anthraquinone derivatives, nidurufin (**188**) and averantin (**189**), isolated from a marine sediment-derived fungus, *P. flavidorsum* SHK1-27, were cytotoxic against K562 cell line (IC_50_ = 12.6 and 27.7 μM, respectively), while adriamycin was used as a positive control with an IC_50_ value of 1.5 μM. Nidurufin (**188**) was suggested to induce cell cycle arrest at the G2/M transition in a time-dependent manner [126]. The total synthesis of (±)-nidurufin (**188**), an aflatoxin precursor, was previously described [127].

**Figure 27:**
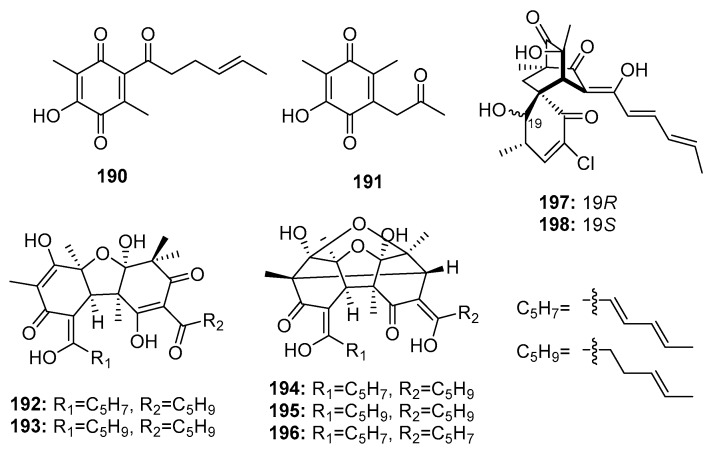
Chemical structures of compounds **190**–**198**.

Members of the sorbicillinoid family are hexaketide metabolites isolated from various fungi. In 2005, Zhu et al. found two sorbicillin analogues (benzoquinone (**190**–**191**)), two bisvertinolones (**192**–**193**), and three bridged bicyclic bisorbicillinoids (**194**–**196**) (Figure 27) in a marine sediment-derived fungus, *P. terrestre*. Dihydrobisvertinolone (**192**) and trichodimerol (**196**) demonstrated the strongest cytotoxic effects (IC_50_ = 0.52 μM in A549, IC_50_ = 0.33 μM in P388, respectively), while etoposide was used as a positive control with IC_50_ values of 1.4 and 0.064 μM, respectively [128,129]. The preliminary SAR showed that an intact sorbyl side chain played a decisive role [130]. Further investigation of this strain yielded two additional chlorinated sorbicillinoids (**197**–**198**). Interestingly, the configuration at C-19 was found to largely determine the cytotoxicity, wherein chloctanspirone A (**197**) (R configuration) was 4-fold more active than chloctanspirone B (**198**) (S configuration) in HL-60 and A549 cancer cell lines [131].

**Figure 28:**
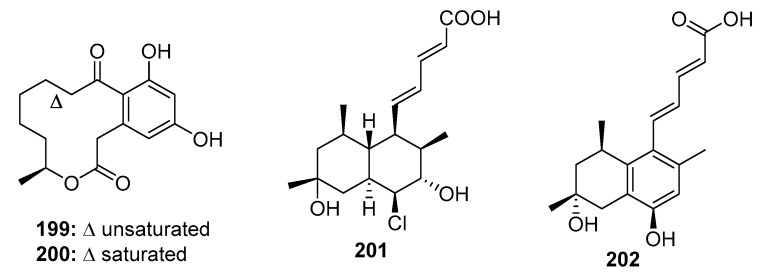
Chemical structures of compounds **199**–**202**.

Macrolides represent a well-known class of antibiotics, and curvularin (**200**) (Figure 28) is a heat shock protein (HSP90) inhibitor [132]. (10*E*, 15*S*)-10,11-Dehydrocurvularin (**199**) was isolated from marine sponge-derived *Penicillium* sp. DRF2 and *Curvularia* sp. It exhibited significant cytotoxicity with mean IC_50_ values ranging from 0.28 to 6 μM in 14 different solid tumors (36 tumor cell lines) [133,134]. *Penicillium* fungi are also a good source of tanzawaic acid polyketides, which exhibit antibiotic resistance [135], as well as anti-inflammatory [136] and cytotoxic activities. Tanzawaic acid P (**201**), isolated from a marine-derived fungus, *Penicillium* sp. CF07370, was selectively toxic to U937 cancer cells via the activation of the mitochondrial apoptotic pathway [137]. Computational ligand-protein-DNA binding analysis revealed that tanzawaic acid D (**202**), isolated from *P. steckii*, effectively and selectively bound to the transcription factor, forkhead box O1 (FOXO1), which can regulate epidermal growth factor receptor (EFGR) signaling, suppress cell cycle progression, and stabilize the conformation of FOXO1-DNA [138].

## 5. Lipopeptides

**Figure 29:**
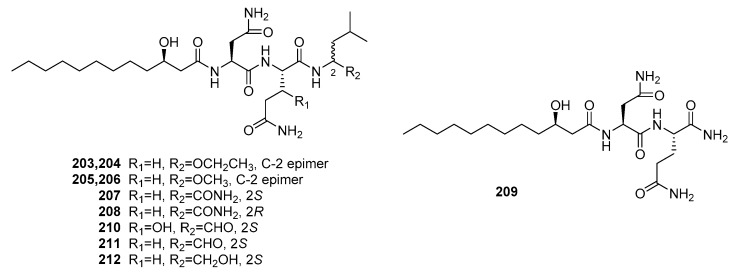
Chemical structures of compounds **203**–**212**.

Fellutamides A and B (**210**–**211**) (Figure 29) were the first cytotoxic lipopeptides isolated from fish-derived *P. fellutanum* [139]. Compounds **210** and **211** exhibited significant cytotoxicity against murine leukemia P388 (IC_50_ = 0.2 and 0.1 μg/mL, respectively), L1210 (IC_50_ = 0.8 and 0.7 μg/mL, respectively), and KB cells (IC_50_ = 0.5 and 0.7 μg/mL, respectively). Recently, seven new similar lipopeptides, penicimutalides A–G (**203**–**209**) and fellutamides B and C (**211**–**212**) were isolated from a diethyl sulfate-induced mutant of the marine fungus, *P. purpurogenum* G59 [140]. They were cytotoxic against five human cancer cell lines (K562, HL-60, Hela, BGC-823, and MCF-7). Compounds **203**–**209** and **212** exhibited weak cytotoxicity (IR% = 10–50% at 100 μg/mL, while 5-fluoruoracil as a positive control with the IR% of 37~50% at 100 μg/mL). However, fellutamide B (**211**) with a C-terminal aldehyde group was more potent with IC_50_ values that ranged from 20 to 80 μg/mL, which indicated that the C-terminal aldehyde group improves the cytotoxicity.

## 6. Miscellaneous Compounds

**Figure 30:**
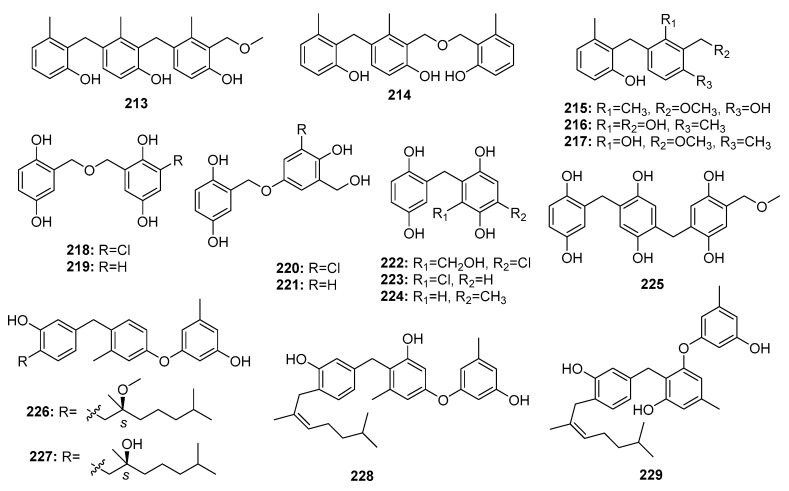
Chemical structures of compounds **213**–**229**.

Polyphenol derivatives are the most abundant fungal secondary metabolites. Unsurprisingly, marine *Penicillium* sp. is a good source of polyphenol derivatives. Two trimeric peniphenylanes A, B (**213**–**214**) and three dimeric peniphenylanes D, F, G (**215**–**217**) (Figure 30) were isolated from the deep sea sediment-derived fungus, *P. fellutanum* HDN14-323. Peniphenylane D (**215**) displayed more potent and extensive cytotoxicity with IC_50_ values in the range of 9–30 μM in three cancer cell lines (Hela, HL-60, and HCT-116), while doxorubicin was used as a positive control with the IC_50_ values of 0.2, 0.6, and 0.2 μM, respectively [141]. The marine sediment-derived fungus, *P. terrestre* was found to produce several gentisyl alcohol derivatives, including trimeric terrestrol A (**225**) and dimeric terrestrols B–H (**218**–**224**), which were found to be cytotoxic against HL-60, MOLT-4, BEL-7402, and A549 cancer cell lines with IC_50_ values in the range of 5–65 μM [142]. Interestingly, the marine mangrove endogenous *P. expansum* 091006 yielded four novel cytotoxic phenolic bisabolane sesquiterpenoids (expansols A–C; E (**226**–**229**)) with IC_50_ values of 15.7, 5.4, 18.2, and 20.8 μM, respectively, in HL-60 cells. In addition, expansol B (**227**) showed significant cytotoxicity against A549 cells (IC_50_ = 1.9 μM), while etoposide was used as a positive control with IC_50_ values of 0.042 and 0.63 μM for two cell lines, respectively [143,144].

**Figure 31:**
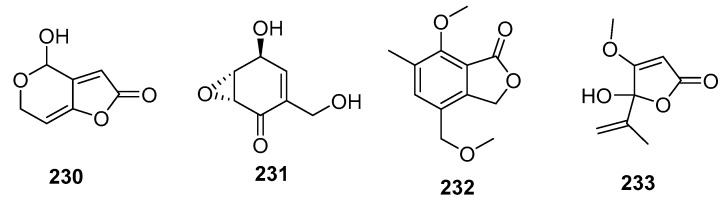
Chemical structures of compounds **230**–**233**.

Patulin (**230**) (Figure 31) is a mycotoxin commonly found in rotting fruits, and is used as a potassium-uptake inhibitor or inducer of ion flux across cell membranes. An alga-derived *Penicillium* sp. was found to produce patulin (**230**) along with (+)-epiepoxydon (**231**), both of which exhibited extraordinary cytotoxic effects in P388 cells (IC_50_ = 0.06 and 0.2 μg/mL, respectively). Furthermore, (+)-epiepoxydon (**231**) had significant cytotoxicity against seven other cancer cell lines with IC_50_ values in the range of 0.3–1.5 μg/mL [111]. The isobenzofurannone derivative (**232**) isolated from a mangrove endophytic *Penicillium* sp. displayed moderate cytotoxicity against KB and KBV200 cells (IC_50_ = 6 and 10 μg/mL, respectively) [145], whereas the penicillic acid (**233**), isolated from marine-derived *Penicillium* strain, exhibited moderate cytotoxicity against POS1, AT6-1(murine prostatic carcinoma), and L929 (murine fibroblasts) cell lines (IC_50_ = 7.8, 29.4, and 12.9 μM, respectively) while doxorubicin was used as a positive control with IC_50_ values of 0.04~2 μM [84].

## 7. Conclusions

The rapid development of marine biotechnology and ever increasing needs of industrial applications resulted in the emergence of marine natural products as alternative drug sources in the early 1990s [146]. Marine-associated microorganisms are sensitive to culture conditions; therefore, strains living in extremely competitive environments tend to provide high potency leads (compound **154** in this review inhibited OVCAR-3 cell line at nanomolar concentrations). Furthermore, the activation of silent gene clusters may activate new biosynthetic pathways that produce compounds with novel structure, which provide equally valid leads (compounds **44** and **45**, which have unique skeletons, had cytotoxic effects in the five cancer cell lines with IC_50_ values of ~10 μM). Interestingly, the halogenation of compound **31**, which was completely inactive, produces compound **32**, which exhibited a much greater potency (compound **32** had significant cytotoxicity in 22Rv1 cells at nanomolar levels) [147].

The genus *Penicillium* has been explored for antitumor leads in recent years [148]. However, the marine ecological diversity of this genus offers more opportunities for drug discovery. This review includes more than 200 cytotoxic or antitumor compounds isolated from marine *Penicillium* fungus and chemically synthesized analogues. Of these, the major metabolites are alkaloids, particularly diketopiperazine alkaloids and indole alkaloids (Appendix A, Table A1). Cytochalasan alkaloids, which are indole alkaloids, constitute a large class of mycotoxins that exhibit significant cytotoxicity against P388 cells (IC_50_ < 1 μg/mL). Furthermore, a series of diketopiperazine alkaloids, gliotoxin analogues, and roquefortine analogues with remarkable cytotoxicity at nanomolar levels are potential anticancer leads. Terpenoid metabolites appear to be more effective against cancer cell lines than steroids; in particular, compounds **99**, **104**, and **132** were effective at nanomolar levels. Furthermore, citrinins (chromone analogues) and their derivatives, which are polyketide mycotoxins, possess excellent cytotoxic activities. Penostatins (cytotoxic polyketides) are cytotoxic to P388 cells with IC_50_ values of ~1 μg/mL. With the exception of **210** and **211**, lipopeptides exhibited moderate cytotoxicity. In addition, the *Penicillium* genus can produce polyphenolic compounds (terrestrols) with pronounced cytotoxicity.

Although our review includes most of the cytotoxic metabolites described in the literature, more compounds are yet to be identified in marine *Penicillium* sp. Different marine hosts and environments can also affect the biosynthesis of metabolites by endozoic fungi. Notably, over 99% of the symbiotic microorganisms cannot be cultured. Further investigations may utilize metagenome libraries of the host organisms to identify more metabolites produced by symbiotic microorganisms [149]. Additionally, further studies are needed to explore the functional mechanisms of the bioactive compounds and to optimize their production.

## Appendix A

**Table A1:** Secondary metabolites from *Penicillium* strain of marine origin. Items are listed according to the metabolite numbers used in this review.

Metabolites	Producing Stain	Environment Source	Type	Cell Lines/Brine Shrimp	IC_50_, LD_50_, or IR (%)	Target	Reference
**Penochalasin A** (**1**)	*Penicillium* sp.	Marine alga	Indole alkaloid	P388	0.4 μg/mL		[8]
**Penochalasin B** (**2**)	*Penicillium* sp.	Marine alga	Indole alkaloid	P388	0.3 μg/mL		[8]
**Penochalasin C** (**3**)	*Penicillium* sp.	Marine alga	Indole alkaloid	P388	0.5 μg/mL		[8]
**Penochalasin D** (**4**)	*Penicillium* sp.	Marine alga	Indole alkaloid	P388	3.2 μg/mL		[7]
**Penochalasin E** (**5**)	*Penicillium* sp.	Marine alga	Indole alkaloid	P388	2.1 μg/mL		[7]
**Penochalasin F** (**6**)	*Penicillium* sp.	Marine alga	Indole alkaloid	P388	1.8 μg/mL		[7]
**Penochalasin G** (**7**)	*Penicillium* sp.	Marine alga	Indole alkaloid	P388	1.9 μg/mL		[7]
**Penochalasin H** (**8**)	*Penicillium* sp.	Marine alga	Indole alkaloid	P388	2.8 μg/mL		[7]
**Penochalasin I** (**9**)	*P. chrysogenum* V11	Mangrove	Indole alkaloid	MDA-MB-435, SGC-7901, A549	(7.55, 7.32, 16.13) μM		[6]
**Penochalasin J** (**10**)	*P. chrysogenum* V11	Mangrove	Indole alkaloid	MDA-MB-435, SGC-7901, A549	(36.68, 37.70, 35.93) μM		[6]
**Chaetoglobosin A** (**11**)	*P. chrysogenum* V11	Mangrove	Indole alkaloid	P388, MDA-MB-435, SGC-7901, A549	0.6 μg/mL (37.56, 7.84, 6.56) μM	Cell-cycle arrest induction, membrane ruffling inhibition, and cell migration	[6,8,9]
**Chaetoglobosin C** (**12**)	*P. chrysogenum* V11	Mangrove	Indole alkaloid	MDA-MB-435, SGC-7901, A549	(19.97, 15.36, 17.82) μM		[6]
**Chaetoglobosin E** (**13**)	*P. chrysogenum* V11	Mangrove	Indole alkaloid	A549	36.63 μM		[6]
**Chaetoglobosin F** (**14**)	*P. chrysogenum* V11	Mangrove	Indole alkaloid	P388, MDA-MB-435, SGC-7901, A549	0.9 μg/mL, (37.77, 26.53, 27.72) μM		[6,8]
**Chaetoglobosin G** (**15**)	*P. chrysogenum* V11	Mangrove	Indole alkaloid	MDA-MB-435, SGC-7901, A549	(38.77, 25.86, 27.63) μM		[6]
**Chaetoglobosin O** (**16**)	*Penicillium* sp.	Marine alga	Indole alkaloid	P388	2.4 μg/mL		[7]
**Cytoglobosin C** (**17**)	*P. chrysogenum* V11	Mangrove	Indole alkaloid	MDA-MB-435, SGC-7901, A549	(12.58, 8.15, 3.35) μM		[6]
**18**	*Penicillium* sp. JMF034	Deep sea sediment	Diketopiperazine	P388	3.4 μM		[12]
**19**	*Penicillium* sp. JMF034	Deep sea sediment	Diketopiperazine	P388	0.058 μM	HMT G9a (IC_50_ = 55 μM)	[12]
**20**	*Penicillium* sp. JMF034	Deep sea sediment	Diketopiperazine	P388	0.11 μM		[12]
**21**	*Penicillium* sp. JMF034	Deep sea sediment	Diketopiperazine	P388	0.11 μM	HMT G9a (IC_50_ = 58 μM)	[12]
**22**	*Penicillium* sp. JMF034	Deep sea sediment	Diketopiperazine	P388	0.056 μM	HMT G9a (IC_50_ = 2.6 μM)	[12]
**Gliotoxin** (**23**)	*Penicillium* sp. JMF034	Deep sea sediment	Diketopiperazine	P388	0.024 μM	HMT G9a (IC_50_ = 6.4 μM) Dual inhibitor of farnesyltransferase and geranylgeranyltransferase I	[12,13]
**Gliotoxin G** (**24**)	*Penicillium* sp. JMF034 *P. brocae* MA-231	Deep sea sediment Mangrove	Diketopiperazine	P388 A2780, A2780 CisR	0.02 μM (0.664, 0.661) μM	HMT G9a (IC_50_ = 2.1 μM)	[12] [14]
**Brozazine A** (**25**)	*P. brocae* MA-231	Mangrove	Diketopiperazine	Du145, Hela, HepG2, MCF-7, NCI-H460, SGC-7901, SW1990, SW480, U251	(4.2, 6.8, 6.4, 5.5, 4.9, 2.6, 6.0, 2.0, 5.2) μM		[16]
**Brozazine B** (**26**)	*P. brocae* MA-231	Mangrove	Diketopiperazine	Du145, Hela, HepG2, MCF-7, NCI-H460, SGC-7901, SW1990, SW480, U251	(3.6, 5.3, 5.5, 6.1, 4.0, 2.4, 6.4, 1.2, 3.5) μM		[16]
**Brozazine E** (**29**)	*P. brocae* MA-231	Mangrove	Diketopiperazine	Du145, Hela, HepG2, MCF-7, NCI-H460, SGC-7901, SW1990, U251	(11.2, 4.3, 5.6, 9.0, 12.4, 3.3, 2.1, 6.1) μM		[16]
**Brozazine F** (**30**)	*P. brocae* MA-231	Mangrove	Diketopiperazine	Du145, Hela, HepG2, MCF-7, NCI-H460, SGC-7901, SW1990, U251	(1.7, 6.9, 2.9, 3.0, 0.89, 8.0, 5.9, 5.3) μM		[16]
***N*-methylpretrichodermam ide B/adametizines A** (**32**)	*P. adametzioides* AS-53 *Penicillium* sp.	Marine sponge/sediment/alga	Diketopiperazine	*Artemia salina*	4.8 μM		[17,18,19]
				L5178Y, 22Rv1, PC-3, LNCaP	(2, 0.51, 5.11, 1.76) μM		
**Roquefortine C** (**33**)	*Penicillium* sp.	Deep sea sediment	Diketopiperazine			Activate *P*-glycoprotein and inhibit P450-3A and other haemoproteins	[20,22]
**Roquefortine F** (**34**)	*Penicillium* sp.	Deep sea sediment	Diketopiperazine	A549, HL-60, BEL-7402, MOLT-4	(14.0, 33.6, 13.0, 21.2) μM		[22]
**Roquefortine G** (**35**)	*Penicillium* sp.	Deep sea sediment	Diketopiperazine	A549, HL-60	(42.5, 36.6) μM		[22]
**Meleagrin** (**38**)	*Penicillium* sp. *P. commune* SD-118	Deep sea sediment	Indole alkaloid	A549, HL-60 HepG2, NCI-H460, Hela, DU145, MDA-MB-231,	(19.9, 7.4) μM (12.0, 22.0, 20.0, 11.0, 5.0) μg/mL	Arrest the cell cycle through G_2_/M phase Inhibitor of tubulin polymerization	[21,22,23]
**Meleagrin B** (**39**)	*Penicillium* sp.	Deep sea sediment	Indole alkaloid	A549, HL-60, BEL-7402, MOLT-4	(2.7, 6.7, 1.8, 2.9) μM		[21,22]
**Meleagrin C** (**40**)	*Penicillium* sp.	Deep sea sediment	Indole alkaloid	A549, BEL-7402, MOLT-4	(9.9, 10.0, 4.7) μM		[22]
**Meleagrin D** (**41**)	*Penicillium* sp.	Deep sea sediment	Indole alkaloid	A549	32.2 μM		[21]
**Meleagrin E** (**42**)	*Penicillium* sp.	Deep sea sediment	Indole alkaloid	A549	55.9 μM		[21]
**Penicimutanin** (**43**)	Mutant *P. purpurogenum* G59	Marine soil	Diketopiperazine	K562, Hela, MCF-7	IR% (100 μg/mL): 22.6%, 17.9%, 26.5%		[24]
**Penicimutanin A** (**44**)	Mutant *P. purpurogenum* G59	Marine soil	Diketopiperazine	K562, HL-60, Hela, BGC-823, MCF-7	(11.4, 5.4, 9.5, 8.0, 5.4) μM		[24]
**Penicimutanin B** (**45**)	Mutant *P. purpurogenum* G59	Marine soil	Diketopiperazine	K562, HL-60, Hela, BGC-823, MCF-7	(19.9, 12.1, 17.7, 16.6, 8.0) μM		[24]
**Fructigenine A** (**46**)	Mutant *P. purpurogenum* G59	Marine soil	Diketopiperazine	K562, Hela, MCF-7, BGC-823	IR% (100μg/mL): 20.8%, 55.3%, 65.6%, 34.8%		[24,25]
**11,11′-dideoxyverticillin A** (**49**)	*Penicillium* sp.	Marine alga	Diketopiperazine	HCT-116	30 ng/mL	Induce G_2_/M arrest through p38 MAPK pathway; Epidermal growth factor receptor tyrosine kinase inhibitor	[28,30,31]
**11′-deoxyverticillin A** (**50**)	*Penicillium* sp.	Marine alga	Diketopiperazine	HCT-116	30 ng/mL		[28]
**Penitrem A** (**51**)	*P. commune isolate* GS20	Sponge/Sediment	Indole alkaloid	MCF, MDA-MB-231 (antiproliferative) MDA-MB-231 (antimigratory) MDA-MB-231 (anti-invasion)	(11.9, 9.8) μM 8.7 μM IR% (15 μM)> 75%	BK channel inhibitor	[34]
**Penitrem B** (**52**)	*P. commune isolate* GS20	Sponge/Sediment	Indole alkaloid	MCF-7, MDA-MB-231 (antiproliferative) MDA-MB-231 (antimigratory)	(5.5, 13.7) μM 10.3 μM		[34]
**Penitrem D** (**53**)	*P. commune isolate* GS20	Sponge/Sediment	Indole alkaloid	MCF-7, MDA-MB-231 (antiproliferative) MDA-MB-231 (antimigratory)	(8.3, 29.7) μM 9.2 μM		[34]
**Penitrem E** (**54**)	*P. commune isolate* GS20	Sponge/Sediment	Indole alkaloid	MCF-7, MDA-MB-231 (antiproliferative) MDA-MB-231 (antimigratory)	(17.5, 25.4) μM 20.3 μM		[34]
**Penitrem F** (**55**)	*P. commune isolate* GS20	Sponge/Sediment	Indole alkaloid	MCF-7, MDA-MB-231 (antiproliferative) MDA-MB-231 (antimigratory)	(15.0, 13.8) μM 35.0 μM		[34]
**6-bromopenitrem B** (**56**)	*P. commune isolate* GS20	Sponge/Sediment	Indole alkaloid	MCF-7, MDA-MB-231 (antiproliferative) MDA-MB-231 (antimigratory) MDA-MB-231 (anti-invasion)	(19.3, 18.8) μM 30.3 μM IR%(15 μM) > 40%		[34]
**6-bromopenitrem E** (**57**)	*P. commune isolate* GS20	Sponge/Sediment	Indole alkaloid	MCF-7, MDA-MB-231 (antiproliferative) MDA-MB-231 (antimigratory)	(16.7, 8.5) μM 9.6 μM	BK channel inhibitor	[34]
**Paspaline** (**58**)	*P. commune isolate* GS20	Sponge/Sediment	Indole alkaloid	MCF-7, MDA-MB-231 (antiproliferative) MDA-MB-231 (antimigratory)	(12.8, 12.4) μM 7.6 μM		[34]
**Emnidole SB** (**59**)	*P. commune isolate* GS20	Sponge/Sediment	Indole alkaloid	MCF-7, MDA-MB-231 (antiproliferative) MDA-MB-231 (antimigratory)	(10.1, 21.3) μM 19.0 μM		[34,37]
**Communesin A** (**60**)	*Penicillium* sp.	Marine alga/Sediment	Indole alkaloid	P388	3.5 μg/mL		[38,40]
**Communesin B** (**61**)	*Penicillium* sp.	Marine alga/Sponge/Sediment	Indole alkaloid	P388, U-937, THP-1, NAMALWA, MOLT-3, SUP-B15	(0.45, 10.4, 11.4, 9.9, 8.1, 7.2) μg/mL		[38,39,40]
**Communesin C** (**62**)	*Penicillium* sp.	Marine sponge	Indole alkaloid	U-937, THP-1, NAMALWA, MOLT-3, SUP-B15	(11.3, 13.1, 8.2, 8.6, 10.8) μg/mL		[39]
**Communesin D** (**63**)	*Penicillium* sp.	Marine sponge	Indole alkaloid	U-937, THP-1, NAMALWA, MOLT-3, SUP-B15	(13.1, 16.2, 14.6, 9.9, 9.0) μg/mL		[39]
**Penioxamide** (**64**)	*P. oxalicum* EN-201	Mangrove	Indole alkaloid	*A. salina*	5.6 μM		[42]
**65**	*P. brefeldianum* SD-273	Marine sediment	Indole alkaloid	*A. salina*	9.4 μM		[43]
**Penipaline B** (**66**)	*P. paneum* SD-44	Marine sediment	Indole alkaloid	A549, HCT-116	(20.44, 14.88) μM		[44]
**Penipaline C** (**67**)	*P. paneum* SD-44	Marine sediment	Indole alkaloid	A549, HCT-116	(21.54, 18.54) μM		[44]
**Terretrione A** (**68**)	*P. vinaceum*	Marine sponge	1,4-diazepane alkaloid	MDA-MB-231	17.7 μM		[45]
**Terretrione C** (**69**)	*Penicillium* sp.CYE-87	Marine tunicate	1,4-diazepane alkaloid	MDA-MB-231	17.6 μM		[46]
**Terretrione D** (**70**)	*Penicillium* sp.CYE-87	Marine tunicate	1,4-diazepane alkaloid	MDA-MB-231	16.5 μM		[46]
**Penicillenol A1** (**71**)	*Penicillium* sp. GQ-7/*P. citrinum*	Mangrove/Marine sediment	Pyrrolidinone alkaloid	A-375, HL-60, A549, BEL-7402, P388	3.2 μg/mL (0.76, 23.8, 13.03, 8.85) μM		[47,49]
**Penicillenol A2** (**72**)	*Penicillium* sp. GQ-7/*P. citrinum*	Mangrove/Marine sediment	Pyrrolidinone alkaloid	A-375 HL-60	13.8 μg/mL 16.26 μM		[47,49]
**Penicillenol B1** (**73**)	*Penicillium* sp. GQ-7/*P. citrinum*	Mangrove/Marine sediment	Pyrrolidinone alkaloid	A-375 HL-60	2.8 μg/mL 3.2 μM		[47,49]
**Penicillenol B2** (**74**)	*Penicillium* sp. GQ-7/*P. citrinum*	Mangrove/Marine sediment	Pyrrolidinone alkaloid	A-375 HL-60	0.97 μg/mL 7.65 μM		[47,49]
**Penicillenol D1** (**75**)	*P. citrinum*	Marine sediment	Pyrrolidinone alkaloid	A549, HL-60	(17.2, 18.5) μg/mL		[48]
**Penicillenol D2** (**76**)	*P. citrinum*	Marine sediment	Pyrrolidinone alkaloid	A549, HL-60	(12.1, 14.5) μg/mL		[48]
**Penitrinine A** (**77**)	*P. citrinum*	Marine sediment	Pyrrolidinone alkaloid	A-375, SPC-A1, HGC-27	(20.12, 28.67, 29.49) μM	Upregulate Bax, downregulate Bcl-2, suppress MMP-9 and TIMP-1	[50]
**78**	*P. janczewskii*	Sea water	Quinolinone	MDA-MB-231, DU-145, SKOV-3, HT-29, A549, CAKI-1, SK-MEL-2, K562	IR % (10 μg/mL) = 20~50%		[52]
**79**	*P. janczewskii*	Sea water	Quinolinone	MDA-MB-231, DU-145, SKOV-3, HT-29, A549, CAKI-1, SK-MEL-2, K562	IR % (10 μg/mL) = 30~90%		[52]
**80**	*P. janczewskii*	Sea water	Quinolinone	MDA-MB-231, DU-145, SKOV-3, HT-29	IR % (10 μg/mL) = 91.6%, 69.2%, 79.8%, 96.0%		[52]
**81**	*Penicillium* sp. ghq208/*Penicillium* sp.	Marine sediment/Mangrove	Quinolinone	95-D, HepG2	(0.57, 6.5) μg/mL		[53,54]
**82**	*Penicillium* sp. ghq208	Marine sediment	Quinolinone	HepG2	13.2 μM		[53]
**83**	*P. commune* SD-118	Deep sea sediment	Quinazolinone	SW1990	20 μg/mL		[23]
**84**	*P. chrysogenum* EN-118	Marine alga	Quinazolinone	DU145, A549, Hela	8 μg/mL		[56]
**85**	*P. oxalicum* 0312F1	Marine (not clear)	Quinazolinone	SGC-7901, BEL-7404	IR % (200 μg/mL) = 30~40%		[55]
**Penipacid A** (**86**)	*P. paneum* SD-44	Deep sea sediment	Amidine alkaloid	RKO	8.4 μM		[57]
**Penipacid E** (**87**)	*P. paneum* SD-44	Deep sea sediment	Amidine alkaloid	RKO	9.7 μM		[57]
**88**	*P. paneum* SD-44	Deep sea sediment	Imine alkaloid	Hela	6.6 μM		[57]
**Penipanoid A** (**89**)	*P. paneum* SD-44	Deep sea sediment	Triazole alkaloid	SMMC-7721	54.2 μM		[58]
**Bis-sclerotioramin** (**90**)	*Penicillium* 303#	Mangrove	Azaphilone alkaloid	MDA-MB-231	7.13 μM		[59]
**Sorbicillactone** (**91**)	*P. chrysogenum*	Marine sponge	Miscellaneous Alkaloid	L5178Y	2.2 μg/mL	Selective anti-leukemic	[60]
**Brocaeloid B** (**92**)	*P. brocae*	Mangrove	Miscellaneous Alkaloid	*A. salina*	36.7 μM		[62]
**Varitatin** (**93**)	Mutant *P. varibile*	Mangrove	Amide alkaloid	HCT-116	2.8 μM	IR%(1μM) = 50% and 40% (PDGFR-βand ErbB4)	[63]
**18-hydroxydecaturin B** (**94**)	*P. oxalicum* EN-201	Mangrove	Pyridinyl-α-pyrone alkaloid	*A. salina*	2.3 μM		[42]
**Xantocillin X** (**95**)	*P. commune* SD-118	Deep sea sediment	Isocyanide alkaloid	MCF-7, HepG2, NCI-H460, Hela, DU145, MDA-MB-231	(12, 7, 10, 10, 8, 8) μg/mL	Inhibit MEK/EPK pathway and activate class III PI3K/Beclin 1 pathway	[23,67]
**96**	*Penicillium* sp. PR19 N-1	Marine sludge	Sesquiterpene	HL-60, A549	(45.8, 82.8) μM		[70]
**97**	*Penicillium* sp. PR19 N-1	Marine sludge	Sesquiterpene	HL-60, A549	(28.3, 5.2) μM		[70]
**98**	*Penicillium* sp. PR19 N-1	Marine sludge	Sesquiterpene	HL-60, A549	(11.8, 12.2) μM		[71]
**99**	*Penicillium* sp. BL 27-2	Sea mud	Sesquiterpene	P388, A549, HL-60, BEL-7402	(0.073, 0.096, 0.065, 4.59) μM		[72]
**Sporogen-AO 1** (**100**)	*Penicillium* sp. BL 27-2	Sea mud	Sesquiterpene	P388, A549, HL-60, BEL-7402	(10.1, 8.81, 10.4, 5.7) μM		[72]
**101**	*Penicillium* sp. BL 27-2	Sea mud	Sesquiterpene	P388, A549, HL-60, BEL-7402	(8.71, 3.51, 7.75, 11.8) μM		[72]
**Adametacorenol B** (**102**)	*P. adametzioides* AS-53	Marine sponge	Diterpene	NCI-H446	5.0 μM		[17]
**Conidiogenone B** (**103**)	*Penicillium* sp.	Deep sea sediment	Diterpene	A549, HL-60	(40.3, 28.2) μM		[22]
**Conidiogenone C** (**104**)	*Penicillium* sp.	Deep sea sediment	Diterpene	HL-60, BEL-7402	(0.038, 0.97) μM		[22]
**Conidiogenone D** (**105**)	*Penicillium* sp.	Deep sea sediment	Diterpene	A549, HL-60, BEL-7402, MOLT-4	(9.3, 5.3, 11.7, 21.1) μM		[22]
**Conidiogenone E** (**106**)	*Penicillium* sp.	Deep sea sediment	Diterpene	A549, HL-60, MOLT-4	(15.1, 8.5, 25.8) μM		[22]
**Conidiogenone F** (**107**)	*Penicillium* sp.	Deep sea sediment	Diterpene	A549, HL-60, BEL-7402	(42.2,17.8, 17.1) μM		[22]
**Conidiogenone G** (**108**)	*Penicillium* sp.	Deep sea sediment	Diterpene	A549, HL-60, BEL-7402, MOLT-4	(8.3, 1.1, 43.2, 4.7) μM		[22]
**Brevione I** (**109**)	*Penicillium* sp.	Deep sea sediment	Diterpene	MCF-7	7.44 μM		[73]
**Brevione A** (**110**)	*Penicillium* sp.	Deep sea sediment	Diterpene	MCF-7	28.4 μM		[73]
**Penicisteroid A** (**111**)	*P. chrysogenum* QEN-24S	Marine alga	Steroid	Hela, SW1990, NCI-H460	(15, 31, 40) μg/mL		[74]
**112**	*Penicillium* sp.	Marine moss	Steroid	HepG2	10.4 μg/mL		[75]
**113**	*Penicillium* sp.	Marine moss	Steroid	HepG2	15.6 μg/mL		[75]
**114**	*Penicillium* sp.	Marine moss	Steroid	HepG2	20.7 μg/mL		[75]
**115**	*Penicillium* sp.	Marine moss	Steroid	HepG2	16.8 μg/mL		[75]
**116**	*Penicillium* sp.	Marine moss	Steroid	HepG2	21.3 μg/mL		[75]
**117**	*P. stoloniferum* QY2-10	Sea squirt	Steroid	P388	4.07 μM		[76]
**118**	*Penicillium* sp.	Marine sponge	Steroid	K562	5.54 μM		[77]
**119**	*Penicillium* sp.	Marine sponge	Steroid	K562	4.38 μM		[77]
**Dankasterone A** (**120**)	*Penicillium* sp.	Marine sponge	Steroid	HL-60, Hela, K562	(0.78, 4.11, 7.57) μM		[77]
**Dankasterone B** (**121**)	*Penicillium* sp.	Marine sponge	Steroid	HL-60, Hela, K562	(3.25, 4.74, 7.89) μM		[77]
**7-deacetoxyyanuthone** (**122**)	*Penicillium* sp.	Marine (not clear)	Meroterpene	A549, SKOV-3, SKMEL-2, XF498, HCT-15	(7.74, 6.35, 3.86, 10.04, 10.07) μg/mL		[79]
**Farnesylbenzenediol** (**123**)	*Penicillium* sp.	Marine (not clear)	Meroterpene	A549, SKOV-3, SKMEL-2, XF498, HCT-15	(4.73, 5.31, 4.80, 5.94, 6.11) μg/mL		[79]
**Farnesylquinone** (**124**)	*Penicillium* sp.	Marine (not clear)	Meroterpene	A549, SKOV-3, SKMEL-2, XF498, HCT-15	(25.44, 37.29, 18.41, 38.07, 42.56) μg/mL		[79]
**Penicillone A** (**125**)	*Penicillium* sp. F11	Marine (not clear)	Meroterpene	HT1080, Cne2	(45.8, 46.2) μM		[80]
**Phenylpyropene E** (**126**)	*P. concentricum* ZLQ-69	Sea water	Sesquiterpene	MGC-803	19.1 μM		[81]
**Phenylpyropene F** (**127**)	*P. concentricum* ZLQ-69	Sea water	Sesquiterpene	MGC-803	13.6 μM		[81]
**Penicillipyrone A** (**128**)	*Penicillium* sp. F446	Marine sediment	Sesquiterpene	K562, A549	(28, 15) μM		[82]
**Penicillipyrone B** (**129**)	*Penicillium* sp. F446	Marine sediment	Sesquiterpene	K562, A549	(50, 17) μM		[82]
**130**	*Penicillium* 303#	Mangrove	Meroterpene	MDA-MB-435, HepG2, HCT-116, A549	(34.25, 24.56, 33.72, 37.82) μg/mL		[59]
**131**	*Penicillium* 303#	Mangrove	Meroterpene	MDA-MB-435, HepG2, HCT-116, A549	(31.32, 23.87, 29.19, 34.06) μg/mL		[59]
**Ligerin** (**132**)	*Penicillium* sp.	Sea water	Merosesquiterpene	POS1, SaOS2, MG63	(117/78, 137, 1459) nM		[84,85]
**Oxalicumone D** (**133**)	*P. oxalicum* SCSGAF 0023	Marine gorgonian	Chromone	BGC823, MOLT-4	(10.10, 5.74) μM		[87]
**Oxalicumone E** (**134**)	*P. oxalicum* SCSGAF 0023	Marine gorgonian	Chromone	H1975, U937, K5652, BGC823, MOLT-4, MCF-7, HL-60, Huh-7	(5.45, 4.16, 8.80, 1.96, 1.36, 4.32, 2.96, 6.33) μM		[87]
**Oxalicumone A** (**135**)	*P. oxalicum* SCSGAF 0023	Marine gorgonian	Chromone	H1975, U937, K5652, BGC823, MOLT-4, MCF-7, HL-60, Huh-7, A375, A549, Hela, HepG2, SW-620, L-02	(10.38, 2.35, 4.53, 4.89, 0.30, 11.30, 2.55, 9.49, 11.7, 41.9, 46.2, 77.8, 22.6, 99.0) μM		[87,88]
**Oxalicumone B** (**136**)	*P. oxalicum* SCSGAF 0023	Marine gorgonian	Chromone	U937, MOLT-4, HL-60, A375, Hela, SW-620	(5.00, 2.30, 6.41, 27.8, 60.9, 40.6) μM		[87,88]
**Chromosulfine** (**145**)	Mutant *P. purpurogenum* G59	Marine (not clear)	Chromone	K562, HL-60, BGC-823, Hela, MCF-7	(60.8, 16.7, 73.8, 75.4, 59.2) μM		[91]
**Secalonic acid F** (**146**)	*Penicillium* sp.	Deep sea sediment	Xanthone	HL-60		Modulate Rho GDP dissociation inhibitor 2 Activate caspase 3 and caspase 9	[92,93]
**Penimethavone A** (**147**)	*P. chrysogenum*	Marine gorgonian	Flavone	Hela, rhabdomyosarcoma	(8.41, 8.18) μM		[94]
**Bipenicillisorin** (**148**)	*P. chrysogenum* SCSIO 41001	Deep sea sediment	Coumarin	K562, A549, Huh-7	(6.78, 6.94, 2.59) μM		[95]
**Monocerin** (**149**)	*Penicillium* sp.	Marine sponge	Coumarin	L5178Y	8.4 μM		[96]
**150**	*Penicillium* sp. ZH16	Mangrove	Coumarin	KB, KBv200	(5, 10) μg/mL		[97]
**Citrinin** (**151**)	*Penicillium* sp FF001	Marine sponge	Azaphilone polyketide			Inhibit respiration complex III	[99,101]
**Penicitrinol L** (**152**)	*P. citrinum*	Marine sediment	Azaphilone polyketide	SW-620	25.6 μM		[48]
**Penicitrinol M** (**153**)	*P. citrinum*	Marine sediment	Azaphilone polyketide	SW-620	20.9 μM		[48]
**Berkelic acid** (**154**)	*Penicillium* sp.	Acid mine lake	Azaphilone polyketide	OVCAR-3 (in NCI60)	91 nM	Inhibit MMP-3 (GI_50_ = 1.87 μM) Inhibit caspase 1 (GI_50_ = 98 μM)	[102]
**Sargassopenilline C** (**155**)	*P. thomii*	Marine alga	Azaphilone polyketide			Inhibit the oncogenic nuclear factor AP-1 (IC_50_ = 15 μM)	[104]
**Sculezonone A** (**156**)	*Penicillium* sp.	Marine sponge	Azaphilone polyketide			Inhibit both DNA polymerases (α and β)	[105]
**Sculezonone B** (**157**)	*Penicillium* sp.	Marine sponge	Azaphilone polyketide			Inhibit both DNA polymerases (α and β)	[105]
**Dicitrinone B** (**158**)	*P. citrinum*	Marine sediment	Azaphilone polyketide			Induce apoptosis through ROS-related caspase pathway	[106]
**Perinadine A** (**159**)	*P. citrinum*	Marine fish	Azaphilone polyketide	L1210	20 μg/mL		[107]
**Herqueiazole** (**160**)	*Penicillium* sp F011	Marine sediment	Azaphilone polyketide	A549	67.3 μM		[108]
**Comazaphilone D** (**161**)	*P. commune* QSD-17	Marine sediment	Azaphilone polyketide	SW1990	51 μM		[109]
**Comazaphilone E** (**162**)	*P. commune* QSD-17	Marine sediment	Azaphilone polyketide	SW1990	26 μM		[109]
**Comazaphilone F** (**163**)	*P. commune* QSD-17	Marine sediment	Azaphilone polyketide	SW1990	53 μM		[109]
**Pinophilin A** (**164**)	*P. pinophilum* Hedgcok	Marine seaweed	Azaphilone polyketide	A549, BALL-1, HCT-116, Hela, NUGC-3	(52.5, 50.2, 51.3, 55.6, 54.7) μM	Inhibit the mammalian DNA polymerases A, B, Y family	[110]
**Pinophilin B** (**165**)	*P. pinophilum* Hedgcok	Marine seaweed	Azaphilone polyketide	A549, BALL-1, HCT-116, Hela, NUGC-3	(93.1, 90.4, 92.5, 99.0, 96.8) μM	Inhibit the mammalian DNA polymerases A, B, Y family	[110]
**Sch 725680** (**166**)	*P. pinophilum* Hedgcok	Marine seaweed	Azaphilone polyketide	A549, BALL-1, HCT-116, Hela, NUGC-3	(65.7, 62.0, 64.6, 68.8, 66.4) μM	Inhibit the mammalian DNA polymerases A, B, Y family	[110]
**Penostatin A** (**167**)	*Penicillium* sp. OUPS-79	Marine alga	Polyketide	P388	0.8 μg/mL	PTP1B inhibitor (IC_50_ = 15.87 μM)	[111,113]
**Penostatin B** (**168**)	*Penicillium* sp. OUPS-79	Marine alga	Polyketide	P388	1.2 μg/mL	PTP1B inhibitor (IC_50_ = 33.65 μM)	[111,113]
**Penostatin C** (**169**)	*Penicillium* sp. OUPS-79	Marine alga	Polyketide	P388, BSY-1, MCF-7, HCC2998, NCI-H522, DMS114, OVCAR-3, MKN1	(1.0, 2.0, 1.6, 2.0, 2.5, 1.9, 2.4, 1.7) μg/mL	PTP1B inhibitor (IC_50_ = 0.37 μM)	[111,113]
**Penostatin D** (**170**)	*Penicillium* sp. OUPS-79	Marine alga	Polyketide	P388	11.0 μg/mL		[111]
**Penostatin E** (**171**)	*Penicillium* sp. OUPS-79	Marine alga	Polyketide	P388	0.9 μg/mL		[111]
**Penostatin F** (**172**)	*Penicillium* sp. OUPS-79	Marine alga	Polyketide	P388	1.4 μg/mL		[112]
**Penostatin G** (**173**)	*Penicillium* sp. OUPS-79	Marine alga	Polyketide	P388	0.5 μg/mL		[112]
**Penostatin H** (**174**)	*Penicillium* sp. OUPS-79	Marine alga	Polyketide	P388	0.8 μg/mL		[112]
**Penostatin I** (**175**)	*Penicillium* sp. OUPS-79	Marine alga	Polyketide	P388	1.2 μg/mL		[112]
**176**	*P. oxalicum* HSY05	Marine sediment	Phenolic polyketide			DNA topoisomerase I inhibitor	[117]
**177**	Co-cultured *Penicillium* sp. WC-29-5	Mangrove	Phenolic polyketide	H1975	3.97 μM		[118]
**178**	Co-cultured *Penicillium* sp. WC-29-5	Mangrove	Phenolic polyketide	H1975, HL-60	(5.73, 3.73) μM		[118]
**(+)-5-chlorogriseofulvin** (**179**)	*P. canescens* MMS460	Sea water	Phenolic polyketide	KB	IR% (0.6 μM) = 49%		[119]
**Griseophenone I** (**180**)	*P. canescens* MMS460	Sea water	Phenolic polyketide	KB	IR% (0.6 μM) = 58%		[119]
**Griseophenone G** (**181**)	*P. canescens* MMS460	Sea water	Phenolic polyketide	KB	IR% (0.6 μM) = 47%		[119]
**Iso-monodictyphenone** (**182**)	*Penicillium* sp. MA-37	Mangrove	Phenolic polyketide	*A. salina*	25.3 μM		[120]
**Penikellide A** (**183**)	*Penicillium* sp. MA-37	Mangrove	Phenolic polyketide	*A. salina*	14.2 μM		[120]
**Penikellide B** (**184**)	*Penicillium* sp. MA-37	Mangrove	Phenolic polyketide	*A. salina*	39.2 μM		[120]
**Penicillide** (**185**)	*Penicillium* sp. ZLN29	Marine sediment	Phenolic polyketide	HepG2	(6.7/9.7, 7.8) μM	ACAT and nonpeptide calpain inhibitor	[121,122,124,125]
**Prepenicillide** (**186**)	*Penicillium* sp. ZLN29	Marine sediment	Phenolic polyketide	HepG2, RD	9.9 μM		[124]
**Hydroxypenicillide** (**187**)	*P. pinophilum*	Marine gorgonian	Phenolic polyketide	Hela	6.1 μM		[125]
**Nidurufin** (**188**)	*P. flavidorsum* SHK1-27	Marine sediment	Anthraquinone	K562	12.6 μM	Induce cell cycle arrest at G_2_/M transition	[126]
**Averantin** (**189**)	*P. flavidorsum* SHK1-27	Marine sediment	Anthraquinone	K562	12.6 μM		[126]
**190**	*P. terrestre*	Marine sediment	Polyketide	A549, P388	(5.3, 15.7) μM		[128]
**191**	*P. terrestre*	Marine sediment	Polyketide	A549, P388	(7.6, 10.5) μM		[128]
**Dihydrobisvertinolone** (**192**)	*P. terrestre*	Marine sediment	Polyketide	A549, P388	(0.52, 1.7) μM		[128]
**193**	*P. terrestre*	Marine sediment	Polyketide	A549	1.4 μM		[128]
**194**	*P. terrestre*	Marine sediment	Polyketide	A549, P388	(2.1, 2.8) μM		[129]
**195**	*P. terrestre*	Marine sediment	Polyketide	A549, P388	(4.3, 8.8) μM		[129]
**Trichodimerol** (**196**)	*P. terrestre*	Marine sediment	Polyketide	A549, P388	(4.7, 0.33) μM		[129]
**Chloctanspirone A** (**197**)	*P. terrestre*	Marine sediment	Polyketide	HL-60, A549	(9.2, 39.7) μM		[131]
**Chloctanspirone B** (**198**)	*P. terrestre*	Marine sediment	Polyketide	HL-60	37.8 μM		[131]
**(10*E*,15*S*)-10,11-Dehydrocurvularin** (**199**)	*Penicillium* sp. DRF2	Marine sponge	Macrolide	36 tumor cell lines	0.28~6 μM		[133,134]
**Curvularin** (**200**)	*Penicillium* sp. DRF2	Marine sponge	Macrolide			HSP90 inhibitor	[132]
**Tanzawaic acid P** (**201**)	*Penicillium* sp. CF07370	Marine sediment	Polyketide	Jurkat, K562, Raji	(28.6, 30.2, 20.3) μM	Active the mitochondrial apoptotic pathway	[137]
**Tanzawaic acid D** (**202**)	*P. steckii*	Marine (not clear)	Polyketide			Bind to the FOXO1 which regulates EFGR signaling and stabilizes the FOXO1-DNA conformation	[138]
**Penicimutamide A** (**203**)	Mutant *P. purpurogenum* G59	Marine soil	Lipopepetide	K562, HL-60, Hela, BGC-823, MCF-7	IR% (100 μg/mL) = 10~40%		[140]
**Penicimutamide B** (**204**)	Mutant *P. purpurogenum* G59	Marine soil	Lipopepetide	K562, HL-60, Hela, BGC-823, MCF-7	IR% (100 μg/mL) = 25~40%		[140]
**Penicimutamide C** (**205**)	Mutant *P. purpurogenum* G59	Marine soil	Lipopepetide	K562, HL-60, Hela, BGC-823, MCF-7	IR% (100 μg/mL) = 10~40%		[140]
**Penicimutamide D** (**206**)	Mutant *P. purpurogenum* G59	Marine soil	Lipopepetide	K562, HL-60, Hela, BGC-823, MCF-7	IR% (100 μg/mL) = 20~40%		[140]
**Penicimutamide E** (**207**)	Mutant *P. purpurogenum* G59	Marine soil	Lipopepetide	K562, HL-60, Hela, BGC-823, MCF-7	IR% (100 μg/mL) = 10~45%		[140]
**Penicimutamide F** (**208**)	Mutant *P. purpurogenum* G59	Marine soil	Lipopepetide	K562, HL-60, Hela, BGC-823, MCF-7	IR% (100 μg/mL) = 10~50%		[140]
**Penicimutamide G** (**209**)	Mutant *P. purpurogenum* G59	Marine soil	Lipopepetide	K562, HL-60, Hela, BGC-823, MCF-7	IR% (100 μg/mL) = 10~20%		[140]
**Fellutamide A** (**210**)	*P. fellutanum*	Marine fish	Lipopepetide	P388, L1210	(0.2, 0.8) μg/mL		[139]
**Fellutamide B** (**211**)	*P. fellutanum*	Marine fish	Lipopepetide	P388, L1210	(0.1, 0.7) μg/mL		[139]
**Fellutamide C** (**212**)	Mutant *P. purpurogenum* G59	Marine soil	Lipopepetide	K562, HL-60, Hela, BGC-823, MCF-7	IR% (100 μg/mL) = 30~50%		[140]
**Peniphenylane A** (**213**)	*P. fellutanum* HDN14-323	Deep sea sediment	Polyphenol	Hela	14.5 μM		[141]
**Peniphenylane B** (**214**)	*P. fellutanum* HDN14-323	Deep sea sediment	Polyphenol	Hela, HCT-116	(11.4, 15.8) μM		[141]
**Peniphenylane D** (**215**)	*P. fellutanum* HDN14-323	Deep sea sediment	Polyphenol	Hela, HL-60, HCT-116	(9.3, 18.2, 31.7) μM		[141]
**Peniphenylane F** (**216**)	*P. fellutanum* HDN14-323	Deep sea sediment	Polyphenol	Hela	29.3 μM		[141]
**Peniphenylane G** (**217**)	*P. fellutanum* HDN14-323	Deep sea sediment	Polyphenol	Hela, HL-60, HCT-116	(16.6, 23.2, 24.7) μM		[141]
**Terrestol B** (**218**)	*P. terrestre*	Marine sediment	Polyphenol	HL-60, MOLT-4, A549, BEL-7402	(6.1, 5.8, 18.3, 62.3) μM		[142]
**Terrestol C** (**219**)	*P. terrestre*	Marine sediment	Polyphenol	HL-60, MOLT-4, A549, BEL-7402	(5.5, 5.6, 18.2, 57.3) μM		[142]
**Terrestol D** (**220**)	*P. terrestre*	Marine sediment	Polyphenol	HL-60, MOLT-4, A549, BEL-7402	(5.3, 5.5, 14.3, 38.5) μM		[142]
**Terrestol E** (**221**)	*P. terrestre*	Marine sediment	Polyphenol	HL-60, MOLT-4, A549, BEL-7402	(54.7, 6.4, 9.6, 59.0) μM		[142]
**Terrestol F** (**222**)	*P. terrestre*	Marine sediment	Polyphenol	HL-60, MOLT-4, A549, BEL-7402	(55.0, 58.1, 13.8, 63.2) μM		[142]
**Terrestol G** (**223**)	*P. terrestre*	Marine sediment	Polyphenol	HL-60, MOLT-4, A549, BEL-7402	(5.1, 6.5, 5.7, 6.0) μM		[142]
**Terrestol H** (**224**)	*P. terrestre*	Marine sediment	Polyphenol	HL-60, MOLT-4, A549, BEL-7402	(6.3, 5.8, 33.8, 61.9) μM		[142]
**Terrestol A** (**225**)	*P. terrestre*	Marine sediment	Polyphenol	HL-60, MOLT-4, A549, BEL-7402	(33.3, 5.5, 23.5, 57.0) μM		[142]
**Expansol A** (**226**)	*P. expansum* 091006	Mangrove	Polyphenol	HL-60	15.7 μM		[143]
**Expansol B** (**227**)	*P. expansum* 091006	Mangrove	Polyphenol	HL-60, A549	(5.4, 1.9) μM		[143,144]
**Expansol C** (**228**)	*P. expansum* 091006	Mangrove	Polyphenol	HL-60	18.2 μM		[143]
**Expansol E** (**229**)	*P. expansum* 091006	Mangrove	Polyphenol	HL-60	20.8 μM		[143]
**Patulin** (**230**)	*Penicillium* sp.	Marine alga	Other	P388, BSY-1, MCF-7, HCC2998, NCI-H522, DMS114, OVCAR-3, MKN1	(0.06, 0.34, 0.65, 1.54, 0.30, 0.57, 0.37, 0.39) μg/mL	Potassium-uptake inhibitor Ion flux across cell membranes inducer	[111]
(**+**)**-Epiepoxydon** (**231**)	*Penicillium* sp.	Marine alga	Other	P388	0.2 μg/mL		[111]
**232**	*Penicillium* sp.	Mangrove	Other	KB, KBv200	(6, 10) μg/mL		[145]
**Penicillic acid** (**233**)	*Penicillium* sp.	Sea water	Other	POS1, AT6-1, L299	(7.8, 29.4, 12.9) μM		[84]
